# Supplementary material for: Validity and reliability of the diagnostic codes for hypochondriasis and dysmorphophobia in the Swedish National Patient Register: a retrospective chart review
Source: BMJ Open. 2021 Dec 5;11(12):e051853. doi: 10.1136/bmjopen-2021-051853 (PMC8650471; doi:10.1136/bmjopen-2021-051853)
Supplement: Supplementary data [file bmjopen-2021-051853supp001.pdf]

Supplementary material

Scoring sheet for the validation of Hypochondriasis and Dysmorphophobia codes

Rater: ____

Participant code: ____ **Sex:** ◯ Man / ◯ Woman

Clinic: ◯ Psychiatry / ◯ Non-psychiatric; specialty: ______________________________________

ICD-10 definition of F45.2 hypochondrical disorder (which includes both hypochondriasis and dysmorphophobia)

Hypochondriacal disorder – The essential feature is a persistent preoccupation with the possibility of having one or more serious and progressive physical disorders. Patients manifest persistent somatic complaints **or** a persistent preoccupation with their physical appearance. Normal or commonplace sensations and appearances are often interpreted by patients as abnormal and distressing, and attention is usually focused upon only one or two organs or systems of the body. Marked depression and anxiety are often present, and may justify additional diagnoses.

Please tick in the right column the code that better describes the clinical presentation:

**Hypochondria**, if persistent somatic complaints	
**Dysmorphophobia**, if persistent preoccupation with their physical appearance	

DSM-IV-TR criteria for Hypochondriasis (tick those that apply on the right column)

**A.** Preoccupation with fears of having, or the idea that one has, a serious disease based on the person's misinterpretation of bodily symptoms.	
**B.** The preoccupation persists despite appropriate medical evaluation and reassurance.	
**C.** The belief in Criterion A is not of delusional intensity (as in Delusional Disorder, Somatic Type) and is not restricted to a circumscribed concern about appearance (as in Body Dysmorphic Disorder).	
**D.** The preoccupation causes clinically significant distress or impairment in social, occupational, or other important areas of functioning.	
**E.** The duration of the disturbance is at least 6 months.	
**F.** The preoccupation is not better accounted for by Generalized Anxiety Disorder, Obsessive-Compulsive Disorder, Panic Disorder, a Major Depressive Episode, Separation Anxiety, or another Somatoform Disorder.	

DSM-5 criteria for Illness Anxiety Disorder (tick those that apply on the right column)

**A.** Preoccupation with having or acquiring a serious illness.	
**B.** Somatic symptoms are not present or, if present, are only mild in intensity. If another medical condition is present or there is a high risk for developing a medical condition (e.g., strong family history is present), the preoccupation is clearly excessive or disproportionate.	

**C.** There is a high level of anxiety about health, and the individual is easily alarmed about personal health status.	
**D.** The individual performs excessive health-related behaviors (e.g., repeatedly checks his or her body for signs of illness) or exhibits maladaptive avoidance (e.g., avoids doctor appointments and hospitals).	
**E.** Illness preoccupation has been present for at least 6 months, but the specific illness that is feared may change over that period of time.	
**F.** The illness-related preoccupation is not better explained by another mental disorder, such as somatic symptom disorder, panic disorder, generalized anxiety disorder, body dysmorphic disorder, obsessive-compulsive disorder, or delusional disorder, somatic type.	

DSM-IV-TR criteria for Body Dysmorphic Disorder (tick those that apply on the right column)

**A.** Preoccupation with an imagined defect in appearance. If a slight physical anomaly is present, the person's concern is markedly excessive.	
**B.** The preoccupation causes clinically significant distress or impairment in social, occupational, or other important areas of functioning.	
**C.** The preoccupation is not better accounted for by another mental disorder (e.g., dissatisfaction with body shape and size in anorexia nervosa).	

DSM-5 criteria for Body Dysmorphic Disorder (tick those that apply on the right column)

**A.** Preoccupation with one or more perceived defects or flaws in physical appearance that are not observable or appear slight to others.	
**B.** At some point during the course of the disorder, the individual has performed repetitive behaviors (e.g., mirror checking, excessive grooming, skin picking, reassurance seeking) or mental acts (e.g., comparing his or her appearance with that of others) in response to the appearance concerns.	
**C.** The preoccupation causes clinically significant distress or impairment in social, occupational, or other important areas of functioning.	
**D.** The appearance preoccupation is not better explained by concerns with body fat or weight in an individual whose symptoms meet diagnostic criteria for an eating disorder.	

Assessment

Diagnosis. Please mark all the answers that apply.

**Hypochondria**	**Body Dysmorphic Disorder**
◯ Diagnostic criteria are clearly met, according to the ICD-10 definition	◯ Diagnostic criteria are clearly met, according to the ICD-10 definition
◯ Diagnostic criteria are clearly met, according to DSM-IV-TR criteria	◯ Diagnostic criteria are clearly met, according to DSM-IV-TR criteria
◯ Diagnostic criteria are clearly met, according to DSM-5 criteria	◯ Diagnostic criteria are clearly met, according to DSM-5 criteria
◯ Probable, according to the ICD-10 definition	◯ Probable, according to the ICD-10 definition
◯ Probable, according to DSM-IV-TR criteria	◯ Probable, according to DSM-IV-TR criteria
◯ Probable, according to DSM-5 criteria	◯ Probable, according to DSM-5 criteria

◯ Neither ICD-10 nor DSM-IV criteria for **Hypochondria** nor **Body Dysmorphic Disorder** are met

◯ Insufficient information to make a decision about either **Hypochondria** or **Body Dysmorphic Disorder** diagnosis because there is no or very minimal description of psychiatric symptoms in the file (e.g., the file comes from a non-psychiatric clinic with no description of psychiatric symptoms, only blood tests available, etc.)

If neither Hypochondria nor Body Dysmorphic Disorder are present, please state the most likely alternative:

____

____

<u>Clinical Global Impression – Severity (CGI-S)</u>. Considering your total clinical experience with this particular population, how mentally ill is the patient? (<u>refer to the hypochondria or body dysmorphic symptoms only; if they are not present, leave this question blank</u>)

0. ◯ Cannot be assessed (insufficient information)
1. ◯ Normal, not at all ill
2. ◯ Borderline mentally ill
3. ◯ Mildly ill
4. ◯ Moderately ill
5. ◯ Markedly ill
6. ◯ Severely ill
7. ◯ Among the most extremely ill patients

<u>Global Assessment of Functioning (GAF) Scale</u>. Consider psychological, social, and occupational functioning on a hypothetical continuum of mental health-illness. Do not include impairment in functioning due to physical (or environmental) limitations.

Please write your score from 0 to 100 *(consult the attached GAF scale for reference)* ☐
