# Supplementary material for: Validity and reliability of the diagnostic codes for hypochondriasis and dysmorphophobia in the Swedish National Patient Register: a retrospective chart review
Source: BMJ Open. 2021 Dec 5;11(12):e051853. doi: 10.1136/bmjopen-2021-051853 (PMC8650471; doi:10.1136/bmjopen-2021-051853)
Supplement: Author's manuscript [file bmjopen-2021-051853.draft_revisions.pdf]

BMJ Open

BMJ Open is committed to open peer review. As part of this commitment we make the peer review history of every article we publish publicly available.

When an article is published we post the peer reviewers' comments and the authors' responses online. We also post the versions of the paper that were used during peer review. These are the versions that the peer review comments apply to.

The versions of the paper that follow are the versions that were submitted during the peer review process. They are not the versions of record or the final published versions. They should not be cited or distributed as the published version of this manuscript.

BMJ Open is an open access journal and the full, final, typeset and author-corrected version of record of the manuscript is available on our site with no access controls, subscription charges or pay-per-view fees (http://bmjopen.bmj.com).

If you have any questions on BMJ Open's open peer review process please email info.bmjopen@bmj.com

BMJ Open

Validity and reliability of the diagnostic codes for hypochondriasis and dysmorphophobia in the Swedish National Patient Register

Journal:	*BMJ Open*
Manuscript ID	bmjopen-2021-051853
Article Type:	Original research
Date Submitted by the Author:	31-Mar-2021
Complete List of Authors:	Rautio, Daniel; Karolinska Institute, Clinical Neuroscience<br>Vilaplana-Pérez, Alba; Karolinska Institute, Department of Clinical Neuroscience<br>Gumpert, Martina; Karolinska Institute, Clinical Neuroscience<br>Ivanov, Volen; Karolinska Institute, Department of Clinical Neuroscience<br>Linde, Johanna; Karolinska Institute, Clinical Neuroscience<br>Österman, Susanna; Karolinska Institute, Clinical Neuroscience<br>Flygare, Oskar; Karolinska Institutet Department of Clinical Neuroscience,<br>Isung, Josef; Karolinska Institute, Clinical Neuroscience<br>Isumura, Kayoko; Karolinska Institute, Clinical Neuroscience<br>Krig, Sonja; Region Stockholm, Stockholm Health Care Services<br>Serlachius , Eva ; Karolinska Institutet,<br>Högström, Jens; Karolinska Institutet Department of Clinical Neuroscience,<br>Rück, Christian; Karolinska Institutet, Department of Clinical Neuroscience<br>Mataix-Cols, David; Karolinska Institute, Department of Clinical Neuroscience<br>Fernández de la Cruz, Lorena; Karolinska Institute, Clinical Neuroscience
Keywords:	Adult psychiatry < PSYCHIATRY, Anxiety disorders < PSYCHIATRY, Child & adolescent psychiatry < PSYCHIATRY

SCHOLARONE™
Manuscripts

I, the Submitting Author has the right to grant and does grant on behalf of all authors of the Work (as defined in the below author licence), an exclusive licence and/or a non-exclusive licence for contributions from authors who are: i) UK Crown employees; ii) where BMJ has agreed a CC-BY licence shall apply, and/or iii) in accordance with the terms applicable for US Federal Government officers or employees acting as part of their official duties; on a worldwide, perpetual, irrevocable, royalty-free basis to BMJ Publishing Group Ltd ("BMJ") its licensees and where the relevant Journal is co-owned by BMJ to the co-owners of the Journal, to publish the Work in this journal and any other BMJ products and to exploit all rights, as set out in our licence.

The Submitting Author accepts and understands that any supply made under these terms is made by BMJ to the Submitting Author unless you are acting as an employee on behalf of your employer or a postgraduate student of an affiliated institution which is paying any applicable article publishing charge ("APC") for Open Access articles. Where the Submitting Author wishes to make the Work available on an Open Access basis (and intends to pay the relevant APC), the terms of reuse of such Open Access shall be governed by a Creative Commons licence – details of these licences and which Creative Commons licence will apply to this Work are set out in our licence referred to above.

Other than as permitted in any relevant BMJ Author's Self Archiving Policies, I confirm this Work has not been accepted for publication elsewhere, is not being considered for publication elsewhere and does not duplicate material already published. I confirm all authors consent to publication of this Work and authorise the granting of this licence.

For peer review only

Word count:

Abstract: 298

Manuscript: 3,295

Tables: 1

Figures: 2

Validity and reliability of the diagnostic codes for hypochondriasis and dysmorphophobia in the Swedish National Patient Register

Daniel Rautio[1,2], Alba Vilaplana-Pérez[1,2], Martina Gumpert[1,2], Volen Z. Ivanov[1,2], Johanna Linde[1], Susanna Österman[1,2], Oskar Flygare[1,2], Josef Isung[1,2], Kayoko Isumura[1,2], Sonja Krig[2], Eva Serlachius[1,2], Jens Högström[1,2], Christian Rück[1,2], David Mataix-Cols[1,2] & Lorena Fernández de la Cruz[1,2]

[1] Centre for Psychiatry Research, Department of Clinical Neuroscience, Karolinska Institutet, Stockholm, Sweden

[2] Stockholm Health Care Services, Region Stockholm, Sweden

Author for correspondence:

Daniel Rautio

Karolinska Institutet, Department of Clinical Neuroscience

Child and Adolescent Psychiatry Research Center

Gävlegatan 22 (Entré B), floor 8

SE-113 30 Stockholm

daniel.rautio@ki.se

For peer review only - http://bmjopen.bmj.com/site/about/guidelines.xhtml

ABSTRACT

Objectives: In the International Classification of Diseases, Tenth Edition (ICD-10), hypochondriasis (illness anxiety disorder) and dysmorphophobia (body dysmorphic disorder) share the same diagnostic code (F45.2). However, the Swedish ICD-10 allows for these disorders to be coded separately (F45.2 and F45.2A, respectively), potentially offering unique opportunities for register-based research on these conditions. We assessed the validity and reliability of their ICD-10 codes in the Swedish National Patient Register (NPR).

Design: Chart review.

Methods: Six hundred individuals with a diagnosis of hypochondriasis or dysmorphophobia (300 each) were randomly selected from the NPR. Their medical files were requested from the corresponding clinics, located anywhere in Sweden. Two independent raters assessed each file according to ICD-10 definitions and Diagnostic and Statistical Manual of Mental Disorders, Fourth Edition, Text Revision (DSM-IV-TR) and Fifth Edition (DSM-5) criteria. Raters also completed the Clinical Global Impression–Severity (CGI-S) and the Global Assessment of Functioning (GAF).

Primary outcome measure: Percent between-rater agreement and positive predictive value (PPV). Intraclass correlation coefficients for the CGI-S and the GAF.

Results: Eighty-four hypochondriasis and 122 dysmorphophobia files were received and analyzed. The inter-rater agreement rate regarding the presence or absence of a diagnosis was 95.2% for hypochondriasis and 92.6% for dysmorphophobia. Sixty-seven hypochondriasis files (79.8%) and 111 dysmorphophobia files (91.0%) were considered 'true positive' cases (PPV=0.80 and PPV=0.91, respectively). CGI-S scores indicated that symptoms were moderately to markedly severe, while GAF scores suggested moderate impairment for

For peer review only - http://bmjopen.bmj.com/site/about/guidelines.xhtml

hypochondriasis and moderate to serious impairment for dysmorphophobia. CGI-S and GAF inter-rater agreement was good for hypochondriasis and moderate for dysmorphophobia.

Conclusions: The Swedish ICD-10 codes for hypochondriasis and dysmorphophobia are sufficiently valid and reliable for register-based studies. The results of such studies should be interpreted in the context of a possible over-representation of severe and highly impaired cases in the register, particularly for dysmorphophobia.

KEYWORDS: Hypochondriasis, illness anxiety disorder, dysmorphophobia, body dysmorphic disorder, validity, reliability, epidemiology.

STRENGTHS AND LIMITATIONS OF THIS STUDY

- Randomly drawn sample of hypochondriasis and dysmorphophobia cases from all over Sweden.
- Thorough review of medical files by at least two independent expert raters.
- Good inter-rater reliability.
- No control diagnostic group.
- Limited number of cases and potential risk of selection bias.

For peer review only

For peer review only - http://bmjopen.bmj.com/site/about/guidelines.xhtml

INTRODUCTION

The National Board of Health and Welfare is a Swedish governmental agency that in 1964 established the National Patient Register (NPR), a health register with individual-level reporting of clinical diagnoses which plays a crucial role in Swedish register-based epidemiological research[1]. The quality of the research conducted on NPR data is highly dependent on the diagnostic validity of the diagnostic codes[2]. Diagnoses in the NPR are coded according to the Swedish International Classification of Diseases (ICD) system, which was adapted from the World Health Organization ICD classification system[1]. The validity of a wide range of ICD diagnostic codes in the Swedish NPR differs between diagnoses, but is generally high[1]. Several diagnostic codes for psychiatric disorders have been examined and generally shown to be sufficiently valid and reliable for research purposes[3-8].

Hypochondriasis (also known as illness anxiety disorder) and dysmophophobia (also known as body dysmorphic disorder) are two chronic and often severe psychiatric disorders associated with significant suffering and a high level of functional impairment[9]. Their estimated prevalence is 1-2% for hypochondriasis[10] and around 2% for dysmorphophobia[11, 12]. In the international version of the ICD-10[13], hypochondriasis and dysmorphophobia are classed as somatoform disorders and share the same diagnostic code (F45.2). It is therefore not possible to separate the two disorders for clinical or research purposes[13]. By contrast, the Swedish version of the ICD-10 includes an additional code that allows clinicians to separately diagnose these two disorders. Specifically, hypochondriasis is coded F45.2, whereas dysmorphophobia is coded F45.2A[14]. This distinction is in line with the most recent classification of these disorders in the ICD-11, which considers them as two separate, but closely related diagnoses within the obsessive-compulsive spectrum[9, 15-18]. Similarly, in the Diagnostic and Statistical Manual of Mental Disorders, Fifth Edition (DSM-5), hypochondriasis (illness anxiety disorder) and dysmorphophobia (body dysmorphic

For peer review only - http://bmjopen.bmj.com/site/about/guidelines.xhtml

disorder) are two different disorders, although they appear under separate chapters (somatoform and obsessive-compulsive and related disorders, respectively)[9, 19, 20]. Thus, the Swedish "anomaly" in the ICD-10 potentially offers a unique opportunity for register-based studies on these disabling psychiatric conditions. However, the validity and reliability of these diagnostic codes has not yet been established.

This study employed a chart review methodology to establish the validity of the Swedish ICD-10 codes for hypochondriasis and dysmorphophobia in the NPR with the aim to assess whether these codes are suitable for future register-based studies.

For peer review only

METHODS

Procedures

After receiving approval from the regional ethical review board in Stockholm (2016/2399-31/5 and 2017/325-32), we requested 600 randomly selected personal identification numbers of individuals who ever received a diagnosis of either hypochondriasis (*n*=300) or dysmorphophobia (*n*=300) from the Swedish National Board of Health and Welfare. In accordance with the protocol approved by the ethical review board, individual patients were not asked for consent, as this would introduce selection biases. No weighting or other adjustments were done to randomly select the cases.

For hypochondriasis, we requested 300 files with the ICD-10 code F45.2 and all its subcodes (except for F45.2A), namely: F45.2B for nosophobia, F45.2C for cancer phobia, F452D for venerophobia, and F45.2X for hypochondriasis, unspecified. For dysmorphophobia, we requested 300 files with the ICD-10 code F45.2A. The dates of registered diagnosis spanned from 1998 to 2016 for those with diagnoses assigned in inpatient clinics, and from 2001 to 2016 for those with diagnoses assigned in outpatient clinics. To be

For peer review only - http://bmjopen.bmj.com/site/about/guidelines.xhtml

eligible for inclusion, a single ICD-10 diagnosis of hypochondriasis or dysmorphophobia at any time during this time period was sufficient.

Following the procedures previously used in other validation studies by the research group[3, 7], once the random cases had been identified, we sent written requests to the corresponding archives or clinics, based on the hospital and medical specialty codes associated to the cases. Cases were excluded when we could not find the associated clinic (e.g., the clinic was no longer operative), when the clinic did not reply or declined participation, when the diagnostic code under study was not documented in the received file or when there was not enough information in the received file to make a diagnostic judgement (e.g., a description of clinical symptoms was not available). **Figure 1** shows the flow for the inclusion of cases for each diagnosis. In total, we received 84 valid cases of hypochondriasis (including 72 cases diagnosed F45.2, hypochondriasis; one case diagnosed F45.2C, cancer phobia; 10 cases diagnosed F45.2X hypochondriasis, unspecified; and one case diagnosed with both F45.2 and F45.2X) and 122 valid cases of dysmorphophobia available for analyses. The length of the received medical records ranged from one to about 1,000 pages.

Chart review

Two raters conducted an independent chart review of each medical record using a predefined scoring sheet (**Supplementary material**). A diagnosis was established independently by each of the two raters, based on all available information in the medical records. The raters for the hypochondriasis files were four clinical psychologists and one psychiatrist, with three of these five raters having a PhD degree. The raters of the dysmorphophobia files were six clinical psychologists and one psychiatrist, three of whom had a PhD. All had extensive clinical experience in the assessment and treatment of their respective disorders.

For peer review only - http://bmjopen.bmj.com/site/about/guidelines.xhtml

Upon revision of the chart, raters decided whether the ICD-10 definition of hypochondriasis or dysmorphophobia was met. Since the ICD-10 contains a narrative description of the disorder, rather than specific operational diagnostic criteria, raters were also asked whether the case under evaluation met diagnostic criteria for the corresponding diagnoses of hypochondriasis or body dysmorphic disorder according to the DSM-IV-TR and, given the recent updates in the diagnostic criteria, also for the DSM-5 illness anxiety disorder or body dysmorphic disorder. If the two independent raters disagreed regarding the presence of a diagnosis, a third blind rater was asked to read the file. In a validation study, the expert rater is considered to be the gold standard and the diagnostic code in the file is the test. Hence, when a rater agreed with the diagnostic code in the file, the case was considered to be a 'true positive', and when a rater considered that a case did not meet criteria for the disorder in question, the case was defined as a 'false positive.' For false positive cases, the raters were asked to provide the most likely alternative diagnosis, according to their clinical judgement.

Since the NPR only includes cases from specialist settings, raters also assessed symptom severity and global functioning related to hypochondriasis or dysmorphophobia in order to better evaluate the representativeness of the cases. These variables were assessed, respectively, by means of the Clinical Global Impression – Severity (CGI-S)[21, 22] and the Global Assessment of Functioning (GAF)[23] rating scales. The CGI-S is a one-item measure assessing the severity of psychopathology from 1 to 7, where 1 is 'normal' and 7 is 'among the most extremely ill patients'[21]. The CGI-S has shown good internal consistency and concurrent validity[24]. The GAF is also a one-item measure (ranging from 1 to 100) used in psychiatry to assess the general social, occupational, and psychological functioning of adults[25]. Scores in the 1-10 range indicate a severely impaired functioning with persistent danger for self or others, whereas scores in the 91-100 range indicate superior functioning with no symptoms. The GAF has shown good validity and reliability in the assessment of

For peer review only - http://bmjopen.bmj.com/site/about/guidelines.xhtml

global functioning in psychiatric patients[25]. Both the CGI-S and the GAF are generally rated in reference to the time of the assessment. Because of the nature of this study, raters were instead asked to make an estimation of the average severity and function of the patient for the whole time covered in the file.

Statistical analyses

The rate of agreement between the two evaluators of each file was calculated. Since the raters' responses in both hypochondriasis and dysmorphophobia cases were very imbalanced (i.e., the answer 'yes' indicating presence of the disorder was much more common for all raters, compared to 'no'), we did not use Kappa statistics to examine inter-rater reliability. This was because in cases with this kind of imbalance in responses, Kappa results may be misleading, showing a paradox where the coefficients are low despite high agreement rates[26, 27]. Instead, we calculated the percent agreement between the two initial raters, which is a valid alternative to Kappa coefficients when using well trained raters who are not likely to guess[28]. The percent agreement is the percent of ratings where both raters made the same judgement.

Further, for each diagnosis, we calculated the positive predictive value (PPV) and their corresponding 95% confidence intervals (CIs). The PPV is calculated by dividing the cases diagnosed correctly by the sum of the true positives and the false positives.

To assess the inter-rater agreement for the CGI-S and the GAF scales, intraclass correlation coefficients (ICC) with 95% CIs were calculated based on one-way mixed-effects model for average measures, absolute agreement[29]. Stata, version 15.1 (StataCorp LLC) was used for all the analyses.

For peer review only - http://bmjopen.bmj.com/site/about/guidelines.xhtml

Patient and public involvement

No patients were involved in setting the research question nor were they involved in developing plans for the study design or data analysis. There are no plans to directly disseminate the results of the research to study participants or the relevant patient community. The dissemination to the public will be achieved through media outreach (for example, press release and communication) upon publication of this study.

For peer review only

RESULTS

Validity and reliability of hypochondriasis codes in the National Patient Register

A total of 84 cases with a register diagnosis of hypochondriasis (45 females, 53.6%) were included in the analysis. The cases came mostly from psychiatric clinics (*n*=75, 89.3%), followed by emergency units (*n*=3, 3.6%), internal medicine clinics (*n*=2, 2.4%), neurology clinics (*n*=2, 2.4%), gynecology clinics (*n*=1, 1.2%), and oncology clinics (*n*=1, 1.2%).

In 80 (95.2%) of the 84 cases, the initial two raters agreed on the presence or absence of a hypochondriasis diagnosis in the file. A third independent rater reviewed the files of four cases where there was a disagreement between the two initial raters: two of these four files were considered true positives and another two were considered false positives.

In total, 67 (79.8%) of the 84 cases were defined as true positives. In the majority of cases (*n*=63, 94%), both raters considered that the criteria were met according to all three diagnostic systems (i.e., ICD-10, DSM-IV-TR, and DSM-5). In the remaining four cases, raters considered that the ICD-10 definition and the DSM-IV-TR diagnostic criteria were met, but not the DSM-5 criteria.

The 67 true positive cases translated into a PPV of 0.80 (95% CI, 0.70-0.88). For the remaining 17 false positive cases, the most frequent alternative diagnosis was

For peer review only - http://bmjopen.bmj.com/site/about/guidelines.xhtml

dysmorphophobia (*n*=11), followed by psychotic disorder (*n*=4), borderline personality disorder (*n*=2), major depressive disorder (*n*=2), somatization disorder (*n*=2), somatoform disorder, unspecified (*n*=2), and obsessive-compulsive disorder (*n*=2) (**Table 1**). Of note, eight of the nine cases from non-psychiatric clinics were considered to be true positives (i.e., correctly classified).

Validity and reliability of dysmorphophobia codes in the National Patient Register

For peer review only

A total of 122 cases with a register diagnosis of dysmorphophobia (83 females, 68.0%) were included in the analysis. The majority of files (*n*=106, 86.9%) came from psychiatric clinics, with the remaining coming from dermatology clinics (*n*=11, 9.0%), plastic surgery clinics (*n*=4, 3.3%), and one from a gynecological clinic (*n*=1, 0.8%).

There was agreement between the two initial raters regarding the presence or absence of a dysmorphophobia diagnosis in 113 of the 122 files (92.6%). Of the nine cases where there was a disagreement, the third independent rater concluded that two were true positives and seven false positives.

In total, 111 (91.0%) of the 122 cases were classed as true positives. In the vast majority of cases (*n*=108, 97.3%), the criteria were met according to all three diagnostic systems, according to both raters. In the three remaining cases, raters considered that the ICD-definition was met, but not all criteria according to the more stringent diagnostic systems, DSM-IV-TR and DSM-5.

Based on the 111 true positive cases, the PPV was 0.91 (95% CI, 0.84-0.95). For the remaining 11 cases defined as false positives, the most frequent alternative diagnoses were excoriation (skin-picking) disorder (*n*=3), factitial dermatitis (*n*=3), eating disorder (*n*=3), hypochondriasis (*n*=2), pervasive development disorder (*n*=2), and psychotic disorder (*n*=2)

For peer review only - http://bmjopen.bmj.com/site/about/guidelines.xhtml

(**Table 1**). Of note, the four cases from plastic surgery clinics were considered to be true positives, as were seven of the eleven cases (63.6%) from dermatology clinics, while the one case from a gynecological clinic was considered to be a false positive.

Severity and global function

CGI-S and the GAF data were available for 63 of the 67 true positive hypochondriasis cases; in the remaining four cases, raters had not scored the scales due to lack of information in the medical file, thus the information was missing. The mean score for the CGI-S was 4.49 (SD=1.01, median=5, interquartile range [IQR]=1) for rater 1 and 4.57 (SD=0.73, median=5, IQR=1) for rater 2, indicating moderate to marked severity of the assessed cases (**Figure 2, panel A**). The inter-rater reliability for the CGI-S was good (ICC=0.75 [95% CI, 0.59-0.85]). The mean GAF score was 54.40 (SD=9.41, median=50, IQR=12) for rater 1 and 52.63 (SD=9.45, median=49, IQR=15) for rater 2, indicating moderate impairment of global functioning (**Figure 2, panel A**). The inter-rater reliability for the GAF was also good (ICC=0.81 [95% CI, 0.69-0.89]).

For dysmorphophobia, CGI-S and GAF scores were available for 94 of the 111 true positive cases; in the remaining 17 cases, raters had not scored the scales due to lack of information in the medical file. The mean score for the CGI-S was 4.70 (SD=1.20, median=4, IQR=2) for rater 1 and 4.99 (SD=0.71, median=5, IQR=1) for rater 2, indicating moderate to marked severity of the assessed cases (**Figure 2, panel B**). The inter-rater reliability for the CGI-S was moderate (ICC=0.61 [95% CI, 0.41-0.74). The mean GAF-score was 47.98 (SD=12.77, median=52.5, IQR=15) as assessed by rater 1 and 47.79 (SD=7.32, median=51, IQR=8) as assessed by rater 2, indicating serious impairment in global functioning (**Figure 2, panel B**). The inter-rater reliability for the GAF was moderate (ICC=0.65 [CI, 0.48-0.77]).

For peer review only - http://bmjopen.bmj.com/site/about/guidelines.xhtml

DISCUSSION

This study evaluated the validity and reliability of the diagnostic codes for hypochondriasis and dysmorphophobia in the Swedish NPR using a chart review design, which is considered to be the gold standard procedure for assessing diagnostic validity[3]. Our results showed that the diagnostic validity of both disorders is generally good, with a PPV of 0.80 for hypochondriasis and 0.91 for dysmorphophobia. These findings are in line with those of previous studies validating other psychiatric disorders in the NPR, including bipolar disorder (PPV=0.81-0.91)[4], schizophrenia (PPV=0.91-1.0)[8], obsessive-compulsive disorder (PPV=0.55-0.96)[3], chronic tic disorders (PPV=0.86-0.97)[3], and social anxiety disorder (PPV=0.72-0.88)[7]. Furthermore, the inter-rater agreement for both hypochondriasis and dysmorphophobia was satisfactory[30].

Nonetheless, 20% of the hypochondriasis files and almost 10% of the dysmorphophobia files were misclassified. For the majority of the misclassified hypochondriasis files (64.7%), dysmorphophobia was suggested as the most likely alternative diagnosis. Since both disorders share the same diagnostic code, it is probable that at least a proportion of those cases were a result of coding errors (i.e., the clinician not knowing that the F45.2A was the corresponding code for dysmorphophobia). In the same way, a smaller but non-negligible proportion of dysmorphophobia cases (18.2%) were judged to better correspond to a diagnosis of hypochondriasis. Thus, it seems that the high proximity and similarity of these adjacent codes poses a challenge for clinicians and may have implications for register-based studies. Because patients receive a new diagnostic code with every specialist visit, individuals in the registers often receive multiple diagnostic codes over time; in this context, it may be wise to question the validity of cases receiving both diagnoses during the follow-up. For this reason, we suggest that future register-based studies using the

For peer review only - http://bmjopen.bmj.com/site/about/guidelines.xhtml

ICD-10 diagnosis of hypochondriasis (F45.2) should exclude individuals with recorded dysmorphophobia codes (F45.2A) at any point during the follow-up, and *vice versa*, in order to reduce the risk of potential misclassification to a minimum.

An additional issue in the register-based epidemiological studies conducted in Sweden is that the NPR only includes diagnoses assigned by physicians in specialist care settings. Therefore, it is often assumed that the patients in the registers are more severe and less functional than the average patient. This may affect the generalizability of the results from register-based studies to non-specialist clinical settings. Contrary to this assumption, the hypochondriasis sample had a broad distribution of severity and global functioning scores, with most patients being moderately ill and having a moderately impaired function. Regarding the dysmorphophobia files, distributions of the severity and functioning variables were somewhat skewed to the more severe end of the spectrum.

It is well known that individuals with hypochondriasis and dysmorphophobia are often reluctant to seek mental health support due to embarrassment about symptoms, poor insight, and a preference for non-psychiatric care (e.g., cosmetic procedures in dysmorphophobia, somatic care in hypochondriasis)[31-34]. As a result, both conditions are presumably severely underdiagnosed[10, 12]. It is therefore paramount to improve detection and diagnosis of these conditions not only amongst mental health practitioners, but also across a wide range of medical specialists (e.g., general practitioners, plastic surgeons, dermatologists). Further, better collaboration between somatic and mental health services is needed in order to improve the chances that these individuals will receive appropriate evidence based treatments.

The main strengths of this study are the random selection of cases from all over Sweden and the thorough review of the medical files by two or three independent expert raters, showing good inter-rater agreement. However, there are also some limitations to

For peer review only - http://bmjopen.bmj.com/site/about/guidelines.xhtml

consider. First, the study had no control diagnostic group, which may result in an increased risk of over-confirming the target diagnosis. Second, there is a risk for selection bias, given that only 28% of the requested hypochondriasis files and 41% of the requested dysmorphophobia files could be included in the final analyses. However, since the reasons for not including the files were mostly practical (e.g., some clinics did no longer exist, had confidentiality concerns or no personnel available to send the files), we assume that a systematic bias is unlikely. Third, we were unable to evaluate the validity of the hypochondriasis subtypes separately given the small number of files received containing these specific codes (11 files containing only the codes F45.2C or F45.2X). Finally, since the raters did not interview the patients in person, the scoring of CGI-S and the GAF should be seen as a general clinical estimate of the patients' severity and general function, rather than a precise assessment.

CONCLUSIONS

The ICD-10 codes for both hypochondriasis and dysmorphophobia in the Swedish NPR are sufficiently valid and reliable for their use in register-based studies. However, the results of such studies should be interpreted in the context of a possible over-representation of severe and highly impaired cases in the register, particularly for dysmorphophobia.

Funding: This study was supported by a grant from Region Stockholm (ALF Medicin grant reference number 20180078) awarded to LFC. AVP was supported by a fellowship from the Alicia Koplowitz Foundation (award/grant number N/A).

For peer review only - http://bmjopen.bmj.com/site/about/guidelines.xhtml

Conflict of interest: DMC receives royalties for contributing articles to UpToDate, Wolters Kluwer Health and Elsevier. LFC receives royalties for contributing articles to UpToDate, Wolters Kluwer Health. The rest of authors declare that they have no competing interests.

Ethics statement: Regional ethical review board in Stockholm (2016/2399-31/5 and 2017/325-32).

Data sharing statement: No additional data are available.

Contributorship statement: LFC, DMC, ES, CR, and JH were involved in the conception of the research question and designed the study protocol. AVP and SK were the data managers and administrators for the project. DR, MG, VZI, JL, SÖ, OF, JI, and KI were independent raters in the chart review. DR contributed to the data management and performed the statistical analyses. DR and LFC drafted the manuscript. LFC and DMC provided supervision. All authors contributed to the final version of the manuscript by providing substantial intellectual contributions. The authors read and approved the final manuscript.

For peer review only - http://bmjopen.bmj.com/site/about/guidelines.xhtml

REFERENCES

1 Ludvigsson JF, Andersson E, Ekbom A, et al. External review and validation of the Swedish national inpatient register. *BMC Public Health* 2011;11:450.
2 Rosen M. National Health Data Registers: a Nordic heritage to public health. *Scand J Public Health* 2002;30(2):81-5.
3 Rück C, Larsson KJ, Lind K, et al. Validity and reliability of chronic tic disorder and obsessive-compulsive disorder diagnoses in the Swedish National Patient Register. *BMJ Open* 2015;5(6):e007520.
4 Sellgren C, Landen M, Lichtenstein P, et al. Validity of bipolar disorder hospital discharge diagnoses: file review and multiple register linkage in Sweden. *Acta Psychiatr Scand* 2011;124(6):447-53.
5 Dalman C, Broms J, Cullberg J, et al. Young cases of schizophrenia identified in a national inpatient register--are the diagnoses valid? *Soc Psychiatry Psychiatr Epidemiol* 2002;37(11):527-31.
6 Idring S, Rai D, Dal H, et al. Autism spectrum disorders in the Stockholm Youth Cohort: design, prevalence and validity. *PLoS One* 2012;7(7):e41280.
7 Vilaplana-Pérez A, Isung J, Krig S, et al. Validity and reliability of social anxiety disorder diagnoses in the Swedish National Patient Register. *BMC Psychiatry* 2020;20(1):242.
8 Ekholm B, Ekholm A, Adolfsson R, et al. Evaluation of diagnostic procedures in Swedish patients with schizophrenia and related psychoses. *Nord J Psychiatry* 2005;59(6):457-64.
9 American Psychiatric Association. The diagnostic and statistical manual of mental disorders. 5 ed: Arlington, VA: Author 2013.
10 Weck F, Richtberg S, Neng J. Epidemiology of Hypochondriasis and Health Anxiety: Comparison of Different Diagnostic Criteria. *Curr Psychiatry Rev* 2014;10.
11 Schneider SC, Turner CM, Mond J, et al. Prevalence and correlates of body dysmorphic disorder in a community sample of adolescents. *Aust N Z J Psychiatry* 2017;51(6):595-603.
12 Veale D, Gledhill LJ, Christodoulou P, et al. Body dysmorphic disorder in different settings: A systematic review and estimated weighted prevalence. *Body Image* 2016;18:168-86.
13 Organization WH. The ICD-10 classification of mental and behavioural disorders: Clinical descriptions and diagnostic guidelines. 10 ed. Geneva: World Health Organization 1992.
14 Socialstyrelsen. Internationell statistisk klassifikation av sjukdomar och relaterade hälsoproblem – Systematisk förteckning, svensk version 2018 (ICD-10-SE). 10 ed 2020.
15 Phillips KA, Wilhelm S, Koran LM, et al. Body dysmorphic disorder: some key issues for DSM-V. *J Depress Anxiety* 2010;27(6):573-91.
16 World Health Organization. International classification of diseases for mortality and morbidity statistics 11 ed 2018.
17 Phillips KA, Stein DJ, Rauch SL, et al. Should an obsessive-compulsive spectrum grouping of disorders be included in DSM-V? *J Depress Anxiety* 2010;27(6):528-55.
18 Stein DJ, Kogan CS, Atmaca M, et al. The classification of Obsessive-Compulsive and Related Disorders in the ICD-11. *J Affect Disord* 2016;190:663-74.
19 DSM-5 Changes: Implications for Child Serious Emotional Disturbance. Table 23, DSM-IV to DSM-5 Body Dysmorphic Disorder Comparison. Substance Abuse and Mental Health Services Administration, Rockville, MD 2016. https://www.ncbi.nlm.nih.gov/books/NBK519712/table/ch3.t19/ (accessed 26 march 2021)
20 Impact of the DSM-IV to DSM-5 Changes on the National Survey on Drug Use and Health. Table 3.32, DSM-IV to DSM-5 Illness Anxiety Disorder Comparison. Substance Abuse and Mental Health Services Administration, Rockville, MD 2016. https://www.ncbi.nlm.nih.gov/books/NBK519704/table/ch3.t32/ (accessed 26 march 2021)
21 Busner J, Targum SD. The clinical global impressions scale: applying a research tool in clinical practice. *Psychiatry (Edgmont)* 2007;4(7):28-37.
22 Guy W. ECDEU assessment manual for psychopharmacology. Rockville, Md: US Dept. of Helath, Education and Welfare, Public Health Service, Alcohol, Drug Abuse and Mental Health Administration, National Institute of Mental Health, Pshychopharmacology, Research Branch, Division of Extramural Reserach Programs 1976.

For peer review only - http://bmjopen.bmj.com/site/about/guidelines.xhtml

23 Endicott J, Spitzer RL, Fleiss JL, et al. The global assessment scale. A procedure for measuring overall severity of psychiatric disturbance. *Arch Gen Psychiatry* 1976;33(6):766-71.
24 Leon AC, Shear MK, Klerman GL, et al. A comparison of symptom determinants of patient and clinician global ratings in patients with panic disorder and depression. *J Clin Psychopharmacol* 1993;13(5):327-31.
25 Jones SH, Thornicroft G, Coffey M, et al. A brief mental health outcome scale-reliability and validity of the Global Assessment of Functioning (GAF). *Br J Psychiatry* 1995;166(5):654-9.
26 Feinstein AR, Cicchetti DV. High agreement but low kappa: I. The problems of two paradoxes. *J Clin Epidemiol* 1990;43(6):543-9.
27 Shankar V, Bangdiwala SI. Observer agreement paradoxes in 2x2 tables: comparison of agreement measures. *BMC Med Res Methodol* 2014;14(1):100.
28 McHugh ML. Interrater reliability: the kappa statistic. *Biochem Med (Zagreb)* 2012;22(3):276-82.
29 Koo TK, Li MY. A Guideline of Selecting and Reporting Intraclass Correlation Coefficients for Reliability Research. *J Chiropr Med* 2016;15(2):155-63.
30 Landis JR, Koch GG. The measurement of observer agreement for categorical data. *Biometrics* 1977;33(1):159-74.
31 Barsky AJ, Ettner SL, Horsky J, et al. Resource utilization of patients with hypochondriacal health anxiety and somatization. *Med Care* 2001;39(7):705-15.
32 Bowyer L, Krebs G, Mataix-Cols D, et al. A critical review of cosmetic treatment outcomes in body dysmorphic disorder. *Body Image* 2016;19:1-8.
33 Phillips K, Didie ER, Menard W, et al. Clinical features of body dysmorphic disorder in adolescents and adults. *Psychiatry Res* 2006;141(3):305-14.
34 Fink P, Ørnbøl E, Christensen KS. The outcome of health anxiety in primary care. A two-year follow-up study on health care costs and self-rated health. *PLoS One* 2010;5(3):e9873.

For peer review only

For peer review only - http://bmjopen.bmj.com/site/about/guidelines.xhtml

TABLES AND FIGURES

Table 1. Alternative diagnoses for false positive cases of hypochondriasis (*n*=17) and dysmorphophobia (*n*=11).

**Hypochondriasis**	***n***	**Dysmorphophobia**	***n***
Dysmorphophobia	11	Excoriation (skin-picking) disorder	3
Psychotic disorder	4	Factitial dermatitis	3
Somatization disorder	2	Eating disorder, unspecified	3
Somatoform disorder, unspecified	2	Hypochondriasis	2
Obsessive-compulsive disorder	2	Psychotic disorder	2
Major depressive disorder	2	Pervasive developmental disorder	2
Borderline personality disorder	2	Delusional disorder	1
General anxiety disorder	1	Somatization disorder	1
Anxiety disorder, unspecified	1	Obsessive-compulsive disorder	1
Bipolar disorder	1	Trichotillomania	1
Pervasive developmental disorder	1	Social phobia	1
Substance dependence disorder	1	Generalized anxiety disorder	1
Acute stress reaction	1	Post-traumatic stress disorder	1
		Gender identity disorder	1
		Borderline personality disorder	1

Note: Numbers do not add up to the total of false positive cases (*n*=17 for hypochondriasis and *n*=11 for dysmorphophobia) since, for multiple cases, raters suggested more than one alternative diagnosis.

For peer review only

For peer review only - http://bmjopen.bmj.com/site/about/guidelines.xhtml

Figure 1. Flowchart of requested and received patient files containing a hypochondriasis (H) or a dysmorphophobia (D) diagnosis code.

Figure 2. Score distribution of the Clinical Global Impression – Severity (CGI-S) and Global Assessment of Functioning (GAF) by rater, for hypochondriasis (Panel A) and for dysmorphophobia (Panel B).

For peer review only

For peer review only - http://bmjopen.bmj.com/site/about/guidelines.xhtml

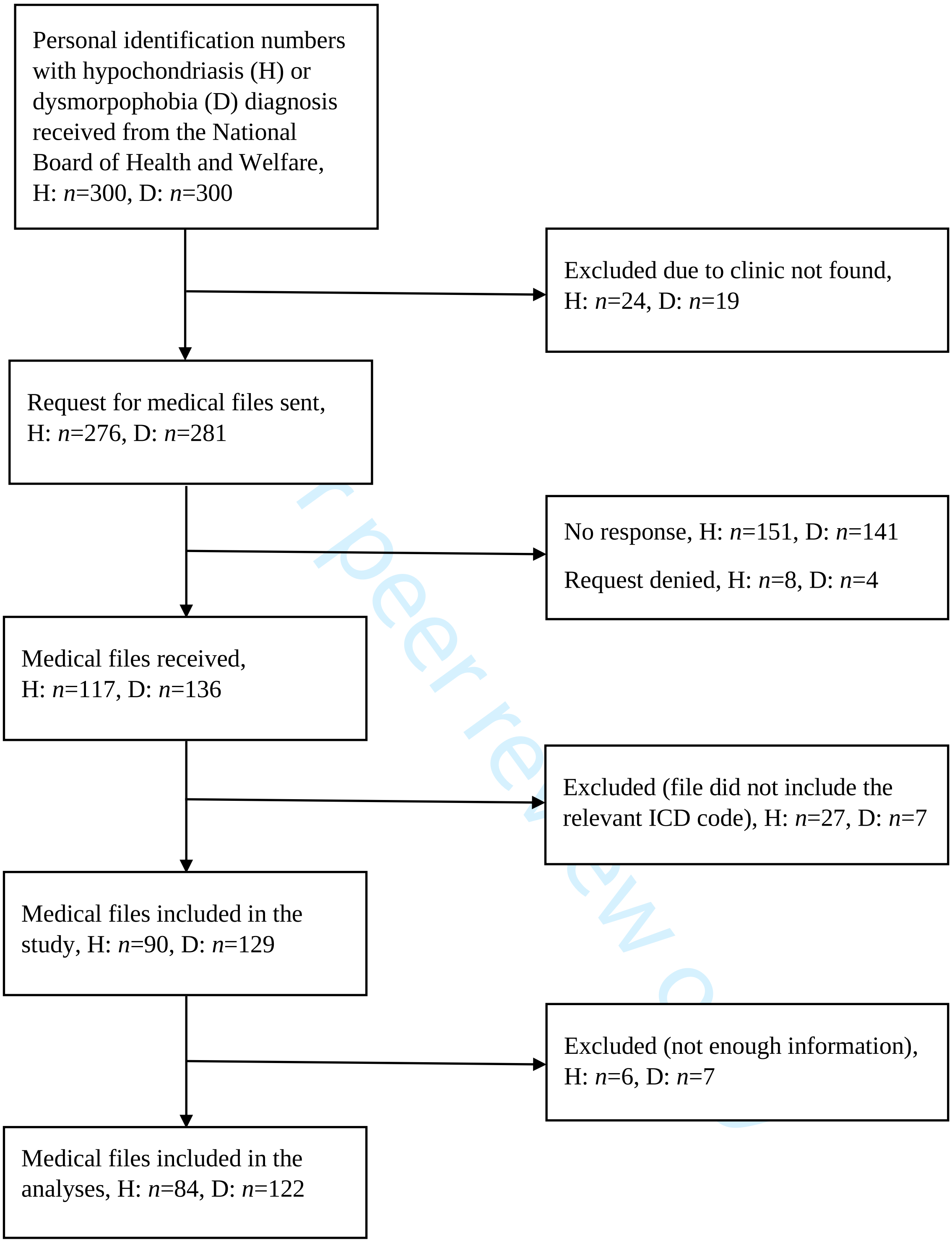

For peer review only - http://bmjopen.bmj.com/site/about/guidelines.xhtml

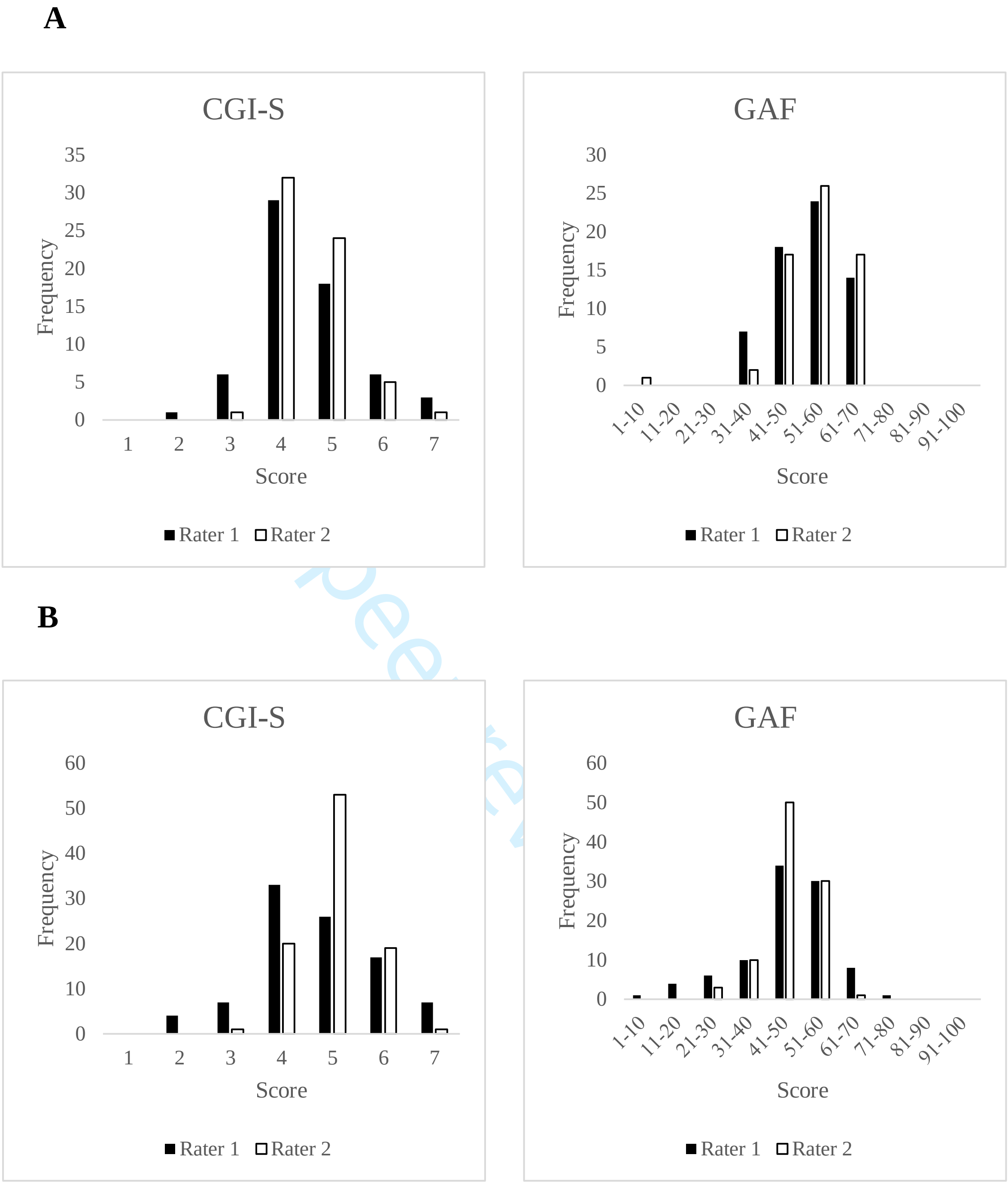
A
CGI-S
Frequency
35
30
25
20
15
10
5
0
1
2
3
4
5
6
7
Score
Rater 1
Rater 2
GAF
Frequency
30
25
20
15
10
5
0
1-10
11-20
21-30
31-40
41-50
51-60
61-70
71-80
81-90
91-100
Score
Rater 1
Rater 2
B
CGI-S
Frequency
60
50
40
30
20
10
0
1
2
3
4
5
6
7
Score
Rater 1
Rater 2
GAF
Frequency
60
50
40
30
20
10
0
1-10
11-20
21-30
31-40
41-50
51-60
61-70
71-80
81-90
91-100
Score
Rater 1
Rater 2

Supplementary material

Scoring sheet for the validation of Hypochondriasis and Dysmorphophobia codes

Rater: ______________________________

Participant code: ______________________________ **Sex:** ◯ Man / ◯ Woman

Clinic: ◯ Psychiatry / ◯ Non-psychiatric; specialty: ______________________________

ICD-10 definition of F45.2 hypochondrical disorder (which includes both hypochondriasis and dysmorphophobia)

Hypochondriacal disorder – The essential feature is a persistent preoccupation with the possibility of having one or more serious and progressive physical disorders. Patients manifest persistent somatic complaints **or** a persistent preoccupation with their physical appearance. Normal or commonplace sensations and appearances are often interpreted by patients as abnormal and distressing, and attention is usually focused upon only one or two organs or systems of the body. Marked depression and anxiety are often present, and may justify additional diagnoses.

Please tick in the right column the code that better describes the clinical presentation:

**Hypochondria**, if persistent somatic complaints	
**Dysmorphophobia**, if persistent preoccupation with their physical appearance	

DSM-IV-TR criteria for Hypochondriasis (tick those that apply on the right column)

**A.** Preoccupation with fears of having, or the idea that one has, a serious disease based on the person's misinterpretation of bodily symptoms.	
**B.** The preoccupation persists despite appropriate medical evaluation and reassurance.	
**C.** The belief in Criterion A is not of delusional intensity (as in Delusional Disorder, Somatic Type) and is not restricted to a circumscribed concern about appearance (as in Body Dysmorphic Disorder).	
**D.** The preoccupation causes clinically significant distress or impairment in social, occupational, or other important areas of functioning.	
**E.** The duration of the disturbance is at least 6 months.	
**F.** The preoccupation is not better accounted for by Generalized Anxiety Disorder, Obsessive-Compulsive Disorder, Panic Disorder, a Major Depressive Episode, Separation Anxiety, or another Somatoform Disorder.	

DSM-5 criteria for Illness Anxiety Disorder (tick those that apply on the right column)

**A.** Preoccupation with having or acquiring a serious illness.	
**B.** Somatic symptoms are not present or, if present, are only mild in intensity. If another medical condition is present or there is a high risk for developing a medical condition (e.g., strong family history is present), the preoccupation is clearly excessive or disproportionate.	

**C.** There is a high level of anxiety about health, and the individual is easily alarmed about personal health status.	
**D.** The individual performs excessive health-related behaviors (e.g., repeatedly checks his or her body for signs of illness) or exhibits maladaptive avoidance (e.g., avoids doctor appointments and hospitals).	
**E.** Illness preoccupation has been present for at least 6 months, but the specific illness that is feared may change over that period of time.	
**F.** The illness-related preoccupation is not better explained by another mental disorder, such as somatic symptom disorder, panic disorder, generalized anxiety disorder, body dysmorphic disorder, obsessive-compulsive disorder, or delusional disorder, somatic type.	

DSM-IV-TR criteria for Body Dysmorphic Disorder (tick those that apply on the right column)

**A.** Preoccupation with an imagined defect in appearance. If a slight physical anomaly is present, the person's concern is markedly excessive.	
**B.** The preoccupation causes clinically significant distress or impairment in social, occupational, or other important areas of functioning.	
**C.** The preoccupation is not better accounted for by another mental disorder (e.g., dissatisfaction with body shape and size in anorexia nervosa).	

DSM-5 criteria for Body Dysmorphic Disorder (tick those that apply on the right column)

**A.** Preoccupation with one or more perceived defects or flaws in physical appearance that are not observable or appear slight to others.	
**B.** At some point during the course of the disorder, the individual has performed repetitive behaviors (e.g., mirror checking, excessive grooming, skin picking, reassurance seeking) or mental acts (e.g., comparing his or her appearance with that of others) in response to the appearance concerns.	
**C.** The preoccupation causes clinically significant distress or impairment in social, occupational, or other important areas of functioning.	
**D.** The appearance preoccupation is not better explained by concerns with body fat or weight in an individual whose symptoms meet diagnostic criteria for an eating disorder.	

Assessment

Diagnosis. Please mark all the answers that apply.

Hypochondria	Body Dysmorphic Disorder
◯ Diagnostic criteria are clearly met, according to the ICD-10 definition	◯ Diagnostic criteria are clearly met, according to the ICD-10 definition
◯ Diagnostic criteria are clearly met, according to DSM-IV-TR criteria	◯ Diagnostic criteria are clearly met, according to DSM-IV-TR criteria
◯ Diagnostic criteria are clearly met, according to DSM-5 criteria	◯ Diagnostic criteria are clearly met, according to DSM-5 criteria
◯ Probable, according to the ICD-10 definition	◯ Probable, according to the ICD-10 definition
◯ Probable, according to DSM-IV-TR criteria	◯ Probable, according to DSM-IV-TR criteria
◯ Probable, according to DSM-5 criteria	◯ Probable, according to DSM-5 criteria

◯ Neither ICD-10 nor DSM-IV criteria for **Hypochondria** nor **Body Dysmorphic Disorder** are met

◯ Insufficient information to make a decision about either **Hypochondria** or **Body Dysmorphic Disorder** diagnosis because there is no or very minimal description of psychiatric symptoms in the file (e.g., the file comes from a non-psychiatric clinic with no description of psychiatric symptoms, only blood tests available, etc.)

If neither Hypochondria nor Body Dysmorphic Disorder are present, please state the most likely alternative:

____

____

Clinical Global Impression – Severity (CGI-S). Considering your total clinical experience with this particular population, how mentally ill is the patient? (refer to the hypochondria or body dysmorphic symptoms only; if they are not present, leave this question blank)

0. ◯ Cannot be assessed (insufficient information)
1. ◯ Normal, not at all ill
2. ◯ Borderline mentally ill
3. ◯ Mildly ill
4. ◯ Moderately ill
5. ◯ Markedly ill
6. ◯ Severely ill
7. ◯ Among the most extremely ill patients

Global Assessment of Functioning (GAF) Scale. Consider psychological, social, and occupational functioning on a hypothetical continuum of mental health-illness. Do not include impairment in functioning due to physical (or environmental) limitations.

Please write your score from 0 to 100 *(consult the attached GAF scale for reference)* ☐

For peer review only

BMJ Open

Validity and reliability of the diagnostic codes for hypochondriasis and dysmorphophobia in the Swedish National Patient Register: A retrospective chart review

Journal:	*BMJ Open*
Manuscript ID	bmjopen-2021-051853.R1
Article Type:	Original research
Date Submitted by the Author:	30-Oct-2021
Complete List of Authors:	Rautio, Daniel; Karolinska Institute, Clinical Neuroscience<br>Vilaplana-Pérez, Alba; Karolinska Institute, Department of Clinical Neuroscience<br>Gumpert, Martina; Karolinska Institute, Clinical Neuroscience<br>Ivanov, Volen; Karolinska Institute, Department of Clinical Neuroscience<br>Linde, Johanna; Karolinska Institute, Clinical Neuroscience<br>Österman, Susanna; Karolinska Institute, Clinical Neuroscience<br>Flygare, Oskar; Karolinska Institutet Department of Clinical Neuroscience,<br>Isung, Josef; Karolinska Institute, Clinical Neuroscience<br>Isumura, Kayoko; Karolinska Institute, Clinical Neuroscience<br>Krig, Sonja; Region Stockholm, Stockholm Health Care Services<br>Serlachius , Eva ; Karolinska Institutet,<br>Högström, Jens; Karolinska Institutet Department of Clinical Neuroscience,<br>Rück, Christian; Karolinska Institutet, Department of Clinical Neuroscience<br>Mataix-Cols, David; Karolinska Institute, Department of Clinical Neuroscience<br>Fernández de la Cruz, Lorena; Karolinska Institute, Clinical Neuroscience
**Primary Subject Heading**:	Mental health
Secondary Subject Heading:	Mental health
Keywords:	Adult psychiatry < PSYCHIATRY, Anxiety disorders < PSYCHIATRY, Child & adolescent psychiatry < PSYCHIATRY

SCHOLARONE™
Manuscripts

I, the Submitting Author has the right to grant and does grant on behalf of all authors of the Work (as defined in the below author licence), an exclusive licence and/or a non-exclusive licence for contributions from authors who are: i) UK Crown employees; ii) where BMJ has agreed a CC-BY licence shall apply, and/or iii) in accordance with the terms applicable for US Federal Government officers or employees acting as part of their official duties; on a worldwide, perpetual, irrevocable, royalty-free basis to BMJ Publishing Group Ltd ("BMJ") its licensees and where the relevant Journal is co-owned by BMJ to the co-owners of the Journal, to publish the Work in this journal and any other BMJ products and to exploit all rights, as set out in our licence.

The Submitting Author accepts and understands that any supply made under these terms is made by BMJ to the Submitting Author unless you are acting as an employee on behalf of your employer or a postgraduate student of an affiliated institution which is paying any applicable article publishing charge ("APC") for Open Access articles. Where the Submitting Author wishes to make the Work available on an Open Access basis (and intends to pay the relevant APC), the terms of reuse of such Open Access shall be governed by a Creative Commons licence – details of these licences and which Creative Commons licence will apply to this Work are set out in our licence referred to above.

Other than as permitted in any relevant BMJ Author's Self Archiving Policies, I confirm this Work has not been accepted for publication elsewhere, is not being considered for publication elsewhere and does not duplicate material already published. I confirm all authors consent to publication of this Work and authorise the granting of this licence.

For peer review only

Word count:

Abstract: 299

Manuscript: 3,249

Tables: 1

Figures: 2

Validity and reliability of the diagnostic codes for hypochondriasis and dysmorphophobia in the Swedish National Patient Register: A retrospective chart review

Daniel Rautio[1,2], Alba Vilaplana-Pérez[1,2], Martina Gumpert[1,2], Volen Z. Ivanov[1,2], Johanna Linde[1], Susanna Österman[1,2], Oskar Flygare[1,2], Josef Isung[1,2], Kayoko Isumura[1,2], Sonja Krig[2], Eva Serlachius[1,2], Jens Högström[1,2], Christian Rück[1,2], David Mataix-Cols[1,2] & Lorena Fernández de la Cruz[1,2]

[1] Centre for Psychiatry Research, Department of Clinical Neuroscience, Karolinska Institutet, Stockholm, Sweden

[2] Stockholm Health Care Services, Region Stockholm, Sweden

Author for correspondence:

Daniel Rautio

Karolinska Institutet, Department of Clinical Neuroscience

Child and Adolescent Psychiatry Research Center

Gävlegatan 22 (Entré B), floor 8

SE-113 30 Stockholm

daniel.rautio@ki.se

For peer review only

ABSTRACT

Objectives: In the International Classification of Diseases, Tenth Edition (ICD-10), hypochondriasis (illness anxiety disorder) and dysmorphophobia (body dysmorphic disorder) share the same diagnostic code (F45.2). However, the Swedish ICD-10 allows for these disorders to be coded separately (F45.2 and F45.2A, respectively), potentially offering unique opportunities for register-based research on these conditions. We assessed the validity and reliability of their ICD-10 codes in the Swedish National Patient Register (NPR).

Design: Retrospective chart review.

Methods: Six hundred individuals with a diagnosis of hypochondriasis or dysmorphophobia (300 each) were randomly selected from the NPR. Their medical files were requested from the corresponding clinics, located anywhere in Sweden. Two independent raters assessed each file according to ICD-10 definitions and Diagnostic and Statistical Manual of Mental Disorders, Fourth Edition, Text Revision (DSM-IV-TR) and Fifth Edition (DSM-5) criteria. Raters also completed the Clinical Global Impression–Severity (CGI-S) and the Global Assessment of Functioning (GAF).

Primary outcome measure: Percent between-rater agreement and positive predictive value (PPV). Intraclass correlation coefficients for the CGI-S and the GAF.

Results: Eighty-four hypochondriasis and 122 dysmorphophobia files were received and analyzed. The inter-rater agreement rate regarding the presence or absence of a diagnosis was 95.2% for hypochondriasis and 92.6% for dysmorphophobia. Sixty-seven hypochondriasis files (79.8%) and 111 dysmorphophobia files (91.0%) were considered 'true positive' cases (PPV=0.80 and PPV=0.91, respectively). CGI-S scores indicated that symptoms were moderately to markedly severe, while GAF scores suggested moderate impairment for

For peer review only - http://bmjopen.bmj.com/site/about/guidelines.xhtml

hypochondriasis and moderate to serious impairment for dysmorphophobia. CGI-S and GAF inter-rater agreement was good for hypochondriasis and moderate for dysmorphophobia.

Conclusions: The Swedish ICD-10 codes for hypochondriasis and dysmorphophobia are sufficiently valid and reliable for register-based studies. The results of such studies should be interpreted in the context of a possible over-representation of severe and highly impaired cases in the register, particularly for dysmorphophobia.

KEYWORDS: Hypochondriasis, illness anxiety disorder, dysmorphophobia, body dysmorphic disorder, validity, reliability, epidemiology.

STRENGTHS AND LIMITATIONS OF THIS STUDY

- Randomly drawn sample of hypochondriasis and dysmorphophobia cases from all over Sweden.
- Thorough review of medical files by at least two independent expert raters.
- Good inter-rater reliability.
- No control diagnostic group.
- Limited number of cases and potential risk of selection bias.

For peer review only

INTRODUCTION

The National Board of Health and Welfare is a Swedish governmental agency that in 1964 established the National Patient Register (NPR), a health register with individual-level reporting of clinical diagnoses which plays a crucial role in Swedish register-based epidemiological research[1]. The quality of the research conducted on NPR data is highly dependent on the diagnostic validity of the diagnostic codes[2]. Diagnoses in the NPR are coded according to the Swedish International Classification of Diseases (ICD) system, which was adapted from the World Health Organization ICD classification system[1]. The validity of a wide range of ICD diagnostic codes in the Swedish NPR differs between diagnoses, but is generally high[1]. Several diagnostic codes for psychiatric disorders have been examined and generally shown to be sufficiently valid and reliable for research purposes[3-8].

Hypochondriasis (also known as illness anxiety disorder) and dysmophophobia (also known as body dysmorphic disorder) are two chronic and often severe psychiatric disorders associated with significant suffering and a high level of functional impairment[9]. Their estimated prevalence is 1-2% for hypochondriasis[10] and around 2% for dysmorphophobia[11, 12]. In the international version of the ICD-10[13], hypochondriasis and dysmorphophobia are classed as somatoform disorders and share the same diagnostic code (F45.2). It is therefore not possible to separate the two disorders for clinical or research purposes[13]. By contrast, the Swedish version of the ICD-10 includes an additional code that allows clinicians to separately diagnose these two disorders. Specifically, hypochondriasis is coded F45.2, whereas dysmorphophobia is coded F45.2A[14]. This distinction is in line with the most recent classification of these disorders in the ICD-11, which considers them as two separate, but closely related diagnoses within the obsessive-compulsive spectrum[9, 15-18]. Similarly, in the Diagnostic and Statistical Manual of Mental Disorders, Fifth Edition (DSM-5), hypochondriasis (illness anxiety disorder) and dysmorphophobia (body dysmorphic

For peer review only - http://bmjopen.bmj.com/site/about/guidelines.xhtml

disorder) are two different disorders, although they appear under separate chapters (somatoform and obsessive-compulsive and related disorders, respectively)[9, 19, 20]. Thus, the Swedish "anomaly" in the ICD-10 potentially offers a unique opportunity for register-based studies on these disabling psychiatric conditions. However, the validity and reliability of these diagnostic codes has not yet been established.

This study employed a chart review methodology to establish the validity of the Swedish ICD-10 codes for hypochondriasis and dysmorphophobia in the NPR with the aim to assess whether these codes are suitable for future register-based studies.

For peer review only

METHODS

Procedures

After receiving approval from the regional ethical review board in Stockholm (2016/2399-31/5 and 2017/325-32), we requested 600 randomly selected personal identification numbers of individuals who ever received a diagnosis of either hypochondriasis (*n*=300) or dysmorphophobia (*n*=300) from the Swedish National Board of Health and Welfare. In accordance with the protocol approved by the ethical review board, individual patients were not asked for consent, as this would introduce selection biases. No weighting or other adjustments were done to randomly select the cases.

For hypochondriasis, we requested 300 files with the ICD-10 code F45.2 and all its subcodes (except for F45.2A), namely: F45.2B for nosophobia, F45.2C for cancer phobia, F452D for venerophobia, and F45.2X for hypochondriasis, unspecified. For dysmorphophobia, we requested 300 files with the ICD-10 code F45.2A. The dates of registered diagnosis spanned from 1998 to 2016 for those with diagnoses assigned in inpatient clinics, and from 2001 to 2016 for those with diagnoses assigned in outpatient clinics. To be

For peer review only - http://bmjopen.bmj.com/site/about/guidelines.xhtml

eligible for inclusion, a single ICD-10 diagnosis of hypochondriasis or dysmorphophobia at any time during this time period was sufficient, regardless of whether the diagnosis was primary or secondary or whether other comorbidities were present.

Following the procedures previously used in other validation studies by the research group[3, 7], once the random cases had been identified, we sent written requests to the corresponding archives or clinics, based on the hospital and medical specialty codes associated to the cases. Cases were excluded when we could not find the associated clinic (e.g., the clinic was no longer operative), when the clinic did not reply or declined participation, when the diagnostic code under study was not documented in the received file or when there was not enough information in the received file to make a diagnostic judgement (e.g., a description of clinical symptoms was not available). **Figure 1** shows the flow for the inclusion of cases for each diagnosis. In total, we received 84 valid cases of hypochondriasis (including 72 cases diagnosed F45.2, hypochondriasis; one case diagnosed F45.2C, cancer phobia; 10 cases diagnosed F45.2X hypochondriasis, unspecified; and one case diagnosed with both F45.2 and F45.2X) and 122 valid cases of dysmorphophobia available for analyses. The length of the received medical records ranged from one to about 1,000 pages.

For peer review only

Chart review

Two raters conducted an independent chart review of each medical record using a predefined scoring sheet (**Supplementary material**). A diagnosis was established independently by each of the two raters, based on all available information in the medical records. The raters for the hypochondriasis files were four clinical psychologists and one psychiatrist, with three of these five raters having a PhD degree. The raters of the dysmorphophobia files were six clinical psychologists and one psychiatrist, three of whom had a PhD. All had extensive clinical experience in the assessment and treatment of their respective disorders.

For peer review only - http://bmjopen.bmj.com/site/about/guidelines.xhtml

Upon revision of the chart, raters decided whether the ICD-10 definition of hypochondriasis or dysmorphophobia was met. Since the ICD-10 contains a narrative description of the disorder, rather than specific operational diagnostic criteria, raters were also asked whether the case under evaluation met diagnostic criteria for the corresponding diagnoses of hypochondriasis or body dysmorphic disorder according to the DSM-IV-TR and, given the recent updates in the diagnostic criteria, also for the DSM-5 illness anxiety disorder or body dysmorphic disorder. If the two independent raters disagreed regarding the presence of a diagnosis, a third blind rater was asked to read the file. In a validation study, the expert rater is considered to be the gold standard and the diagnostic code in the file is the test. Hence, when a rater agreed with the diagnostic code in the file, the case was considered to be a 'true positive', and when a rater considered that a case did not meet criteria for the disorder in question, the case was defined as a 'false positive.' For false positive cases, the raters were asked to provide the most likely alternative diagnosis, according to their clinical judgement.

Since the NPR only includes cases from specialist settings, raters also assessed symptom severity and global functioning related to hypochondriasis or dysmorphophobia in order to better evaluate the representativeness of the cases. These variables were assessed, respectively, by means of the Clinical Global Impression – Severity (CGI-S)[21, 22] and the Global Assessment of Functioning (GAF)[23] rating scales. The CGI-S is a one-item measure assessing the severity of psychopathology from 1 to 7, where 1 is 'normal' and 7 is 'among the most extremely ill patients'[21]. The CGI-S has shown good internal consistency and concurrent validity[24]. The GAF is also a one-item measure (ranging from 1 to 100) used in psychiatry to assess the general social, occupational, and psychological functioning of adults[25]. Scores in the 1-10 range indicate a severely impaired functioning with persistent danger for self or others, whereas scores in the 91-100 range indicate superior functioning with no symptoms. The GAF has shown good validity and reliability in the assessment of

For peer review only - http://bmjopen.bmj.com/site/about/guidelines.xhtml

global functioning in psychiatric patients[25]. Both the CGI-S and the GAF are generally rated in reference to the time of the assessment. Because of the nature of this study, raters were instead asked to make an estimation of the average severity and function of the patient for the whole time covered in the file.

Statistical analyses

The rate of agreement between the two evaluators of each file was calculated. Since the raters' responses in both hypochondriasis and dysmorphophobia cases were very imbalanced (i.e., the answer 'yes' indicating presence of the disorder was much more common for all raters, compared to 'no'), we did not use Kappa statistics to examine inter-rater reliability. This was because in cases with this kind of imbalance in responses, Kappa results may be misleading, showing a paradox where the coefficients are low despite high agreement rates[26, 27]. Instead, we calculated the percent agreement between the two initial raters, which is a valid alternative to Kappa coefficients when using well trained raters who are not likely to guess[28]. The percent agreement is the percent of ratings where both raters made the same judgement.

Further, for each diagnosis, we calculated the positive predictive value (PPV) and their corresponding 95% confidence intervals (CIs). The PPV is calculated by dividing the cases diagnosed correctly by the sum of the true positives and the false positives.

To assess the inter-rater agreement for the CGI-S and the GAF scales, intraclass correlation coefficients (ICC) with 95% CIs were calculated based on one-way mixed-effects model for average measures, absolute agreement[29]. Stata, version 15.1 (StataCorp LLC) was used for all the analyses.

For peer review only - http://bmjopen.bmj.com/site/about/guidelines.xhtml

Patient and public involvement

No patients were involved in setting the research question nor were they involved in developing plans for the study design or data analysis. There are no plans to directly disseminate the results of the research to study participants or the relevant patient community. The dissemination to the public will be achieved through media outreach (for example, press release and communication) upon publication of this study.

For peer review only

RESULTS

Validity and reliability of hypochondriasis codes in the National Patient Register

A total of 84 cases with a register diagnosis of hypochondriasis (45 females, 53.6%) were included in the analysis. The cases came mostly from psychiatric clinics (*n*=75, 89.3%), followed by emergency units (*n*=3, 3.6%), internal medicine clinics (*n*=2, 2.4%), neurology clinics (*n*=2, 2.4%), gynecology clinics (*n*=1, 1.2%), and oncology clinics (*n*=1, 1.2%).

In 80 (95.2%) of the 84 cases, the initial two raters agreed on the presence or absence of a hypochondriasis diagnosis in the file. A third independent rater reviewed the files of four cases where there was a disagreement between the two initial raters: two of these four files were considered true positives and another two were considered false positives.

In total, 67 (79.8%) of the 84 cases were defined as true positives. In the majority of cases (*n*=63, 94%), both raters considered that the criteria were met according to all three diagnostic systems (i.e., ICD-10, DSM-IV-TR, and DSM-5). In the remaining four cases, raters considered that the ICD-10 definition and the DSM-IV-TR diagnostic criteria were met, but not the DSM-5 criteria.

The 67 true positive cases translated into a PPV of 0.80 (95% CI, 0.70-0.88). For the remaining 17 false positive cases, the most frequent alternative diagnosis was

For peer review only - http://bmjopen.bmj.com/site/about/guidelines.xhtml

dysmorphophobia (*n*=11), followed by psychotic disorder (*n*=4), borderline personality disorder (*n*=2), major depressive disorder (*n*=2), somatization disorder (*n*=2), somatoform disorder, unspecified (*n*=2), and obsessive-compulsive disorder (*n*=2) (**Table 1**). Of note, eight of the nine cases from non-psychiatric clinics were considered to be true positives (i.e., correctly classified).

Validity and reliability of dysmorphophobia codes in the National Patient Register

A total of 122 cases with a register diagnosis of dysmorphophobia (83 females, 68.0%) were included in the analysis. The majority of files (*n*=106, 86.9%) came from psychiatric clinics, with the remaining coming from dermatology clinics (*n*=11, 9.0%), plastic surgery clinics (*n*=4, 3.3%), and one from a gynecological clinic (*n*=1, 0.8%).

There was agreement between the two initial raters regarding the presence or absence of a dysmorphophobia diagnosis in 113 of the 122 files (92.6%). Of the nine cases where there was a disagreement, the third independent rater concluded that two were true positives and seven false positives.

In total, 111 (91.0%) of the 122 cases were classed as true positives. In the vast majority of cases (*n*=108, 97.3%), the criteria were met according to all three diagnostic systems, according to both raters. In the three remaining cases, raters considered that the ICD-definition was met, but not all criteria according to the more stringent diagnostic systems, DSM-IV-TR and DSM-5.

Based on the 111 true positive cases, the PPV was 0.91 (95% CI, 0.84-0.95). For the remaining 11 cases defined as false positives, the most frequent alternative diagnoses were excoriation (skin-picking) disorder (*n*=3), factitial dermatitis (*n*=3), eating disorder (*n*=3), hypochondriasis (*n*=2), pervasive development disorder (*n*=2), and psychotic disorder (*n*=2)

For peer review only - http://bmjopen.bmj.com/site/about/guidelines.xhtml

(**Table 1**). Of note, the four cases from plastic surgery clinics were considered to be true positives, as were seven of the eleven cases (63.6%) from dermatology clinics, while the one case from a gynecological clinic was considered to be a false positive.

Severity and global function

CGI-S and the GAF data were available for 63 of the 67 true positive hypochondriasis cases; in the remaining four cases, raters had not scored the scales due to lack of information in the medical file, thus the information was missing. The mean score for the CGI-S was 4.49 (SD=1.01, median=5, interquartile range [IQR]=1) for rater 1 and 4.57 (SD=0.73, median=5, IQR=1) for rater 2, indicating moderate to marked severity of the assessed cases (**Figure 2, panel A**). The inter-rater reliability for the CGI-S was good (ICC=0.75 [95% CI, 0.59-0.85]). The mean GAF score was 54.40 (SD=9.41, median=50, IQR=12) for rater 1 and 52.63 (SD=9.45, median=49, IQR=15) for rater 2, indicating moderate impairment of global functioning (**Figure 2, panel A**). The inter-rater reliability for the GAF was also good (ICC=0.81 [95% CI, 0.69-0.89]).

For dysmorphophobia, CGI-S and GAF scores were available for 94 of the 111 true positive cases; in the remaining 17 cases, raters had not scored the scales due to lack of information in the medical file. The mean score for the CGI-S was 4.70 (SD=1.20, median=4, IQR=2) for rater 1 and 4.99 (SD=0.71, median=5, IQR=1) for rater 2, indicating moderate to marked severity of the assessed cases (**Figure 2, panel B**). The inter-rater reliability for the CGI-S was moderate (ICC=0.61 [95% CI, 0.41-0.74). The mean GAF-score was 47.98 (SD=12.77, median=52.5, IQR=15) as assessed by rater 1 and 47.79 (SD=7.32, median=51, IQR=8) as assessed by rater 2, indicating serious impairment in global functioning (**Figure 2, panel B**). The inter-rater reliability for the GAF was moderate (ICC=0.65 [CI, 0.48-0.77]).

For peer review only - http://bmjopen.bmj.com/site/about/guidelines.xhtml

DISCUSSION

This study evaluated the validity and reliability of the diagnostic codes for hypochondriasis and dysmorphophobia in the Swedish NPR using a chart review design, which is considered to be the gold standard procedure for assessing diagnostic validity[3]. Our results showed that the diagnostic validity of both disorders is generally good, with a PPV of 0.80 for hypochondriasis and 0.91 for dysmorphophobia. These findings are in line with those of previous studies validating other psychiatric disorders in the NPR, including bipolar disorder (PPV=0.81-0.91)[4], schizophrenia (PPV=0.91-1.0)[8], obsessive-compulsive disorder (PPV=0.55-0.96)[3], chronic tic disorders (PPV=0.86-0.97)[3], and social anxiety disorder (PPV=0.72-0.88)[7]. Furthermore, the inter-rater agreement for both hypochondriasis and dysmorphophobia was satisfactory[30].

Nonetheless, 20% of the hypochondriasis files and almost 10% of the dysmorphophobia files were misclassified. For the majority of the misclassified hypochondriasis files (64.7%), dysmorphophobia was suggested as the most likely alternative diagnosis. Since both disorders share the same diagnostic code, it is probable that at least a proportion of those cases were a result of coding errors (i.e., the clinician not knowing that the F45.2A was the corresponding code for dysmorphophobia). In the same way, a smaller but non-negligible proportion of dysmorphophobia cases (18.2%) were judged to better correspond to a diagnosis of hypochondriasis. Thus, it seems that the high proximity and similarity of these adjacent codes poses a challenge for clinicians and may have implications for register-based studies. Because patients receive a new diagnostic code with every specialist visit, individuals in the registers often receive multiple diagnostic codes over time; in this context, it may be wise to question the validity of cases receiving both diagnoses during the follow-up. For this reason, we suggest that future register-based studies using the

For peer review only - http://bmjopen.bmj.com/site/about/guidelines.xhtml

ICD-10 diagnosis of hypochondriasis (F45.2) should exclude individuals with recorded dysmorphophobia codes (F45.2A) at any point during the follow-up, and *vice versa*, in order to reduce the risk of potential misclassification to a minimum.

An additional issue in the register-based epidemiological studies conducted in Sweden is that the NPR only includes diagnoses assigned by physicians in specialist care settings. Further, it is well known that individuals with hypochondriasis and dysmorphophobia are often reluctant to seek mental health support due to embarrassment about symptoms, poor insight, and a preference for non-psychiatric care (e.g., cosmetic procedures in dysmorphophobia, somatic care in hypochondriasis)[31-34]. As a result, both conditions are presumably severely underdiagnosed[10, 12] and it could be assumed that the patients in the registers are more severe and less functional than the average patient. This may affect the generalizability of the results from register-based studies to non-specialist clinical settings. Nonetheless, the hypochondriasis sample had a broad distribution of severity and global functioning scores, with most patients being moderately ill and having a moderately impaired function. Regarding the dysmorphophobia files, distributions of the severity and functioning variables were somewhat skewed to the more severe end of the spectrum.

The main strengths of this study are the random selection of cases from all over Sweden and the thorough review of the medical files by two or three independent expert raters, showing good inter-rater agreement. However, there are also some limitations to consider. First, the study had no control diagnostic group, which may result in an increased risk of over-confirming the target diagnosis. Second, there is a risk for selection bias, given that only 28% of the requested hypochondriasis files and 41% of the requested dysmorphophobia files could be included in the final analyses. However, since the reasons for not including the files were mostly practical (e.g., some clinics did no longer exist, had

For peer review only - http://bmjopen.bmj.com/site/about/guidelines.xhtml

confidentiality concerns or no personnel available to send the files), we assume that a systematic bias is unlikely. Third, we were unable to evaluate the validity of the hypochondriasis subtypes separately given the small number of files received containing these specific codes (11 files containing only the codes F45.2C or F45.2X). Finally, since the raters did not interview the patients in person, the scoring of CGI-S and the GAF should be seen as a general clinical estimate of the patients' severity and general function, rather than a precise assessment.

CONCLUSIONS

The ICD-10 codes for both hypochondriasis and dysmorphophobia in the Swedish NPR are sufficiently valid and reliable for their use in register-based studies. However, the results of such studies should be interpreted in the context of a possible over-representation of severe and highly impaired cases in the register, particularly for dysmorphophobia.

Funding: This study was supported by a grant from Region Stockholm (ALF Medicin grant reference number 20180078) awarded to LFC. AVP was supported by a fellowship from the Alicia Koplowitz Foundation (award/grant number N/A).

Conflict of interest: DMC receives royalties for contributing articles to UpToDate, Wolters Kluwer Health and Elsevier. LFC receives royalties for contributing articles to UpToDate, Wolters Kluwer Health. The rest of authors declare that they have no competing interests.

For peer review only

For peer review only - http://bmjopen.bmj.com/site/about/guidelines.xhtml

Ethics statement: Regional ethical review board in Stockholm (2016/2399-31/5 and 2017/325-32).

Data sharing statement: No additional data are available.

Contributorship statement: LFC, DMC, ES, CR, and JH were involved in the conception of the research question and designed the study protocol. AVP and SK were the data managers and administrators for the project. DR, MG, VZI, JL, SÖ, OF, JI, and KI were independent raters in the chart review. DR contributed to the data management and performed the statistical analyses. DR and LFC drafted the manuscript. LFC and DMC provided supervision. All authors contributed to the final version of the manuscript by providing substantial intellectual contributions. The authors read and approved the final manuscript.

For peer review only

REFERENCES

1 Ludvigsson JF, Andersson E, Ekbom A, et al. External review and validation of the Swedish national inpatient register. *BMC Public Health* 2011;11:450.
2 Rosen M. National Health Data Registers: a Nordic heritage to public health. *Scand J Public Health* 2002;30(2):81-5.
3 Rück C, Larsson KJ, Lind K, et al. Validity and reliability of chronic tic disorder and obsessive-compulsive disorder diagnoses in the Swedish National Patient Register. *BMJ Open* 2015;5(6):e007520.
4 Sellgren C, Landen M, Lichtenstein P, et al. Validity of bipolar disorder hospital discharge diagnoses: file review and multiple register linkage in Sweden. *Acta Psychiatr Scand* 2011;124(6):447-53.
5 Dalman C, Broms J, Cullberg J, et al. Young cases of schizophrenia identified in a national inpatient register--are the diagnoses valid? *Soc Psychiatry Psychiatr Epidemiol* 2002;37(11):527-31.
6 Idring S, Rai D, Dal H, et al. Autism spectrum disorders in the Stockholm Youth Cohort: design, prevalence and validity. *PLoS One* 2012;7(7):e41280.
7 Vilaplana-Pérez A, Isung J, Krig S, et al. Validity and reliability of social anxiety disorder diagnoses in the Swedish National Patient Register. *BMC Psychiatry* 2020;20(1):242.
8 Ekholm B, Ekholm A, Adolfsson R, et al. Evaluation of diagnostic procedures in Swedish patients with schizophrenia and related psychoses. *Nord J Psychiatry* 2005;59(6):457-64.
9 American Psychiatric Association. The diagnostic and statistical manual of mental disorders. 5 ed: Arlington, VA: Author 2013.
10 Weck F, Richtberg S, Neng J. Epidemiology of Hypochondriasis and Health Anxiety: Comparison of Different Diagnostic Criteria. *Curr Psychiatry Rev* 2014;10.
11 Schneider SC, Turner CM, Mond J, et al. Prevalence and correlates of body dysmorphic disorder in a community sample of adolescents. *Aust N Z J Psychiatry* 2017;51(6):595-603.
12 Veale D, Gledhill LJ, Christodoulou P, et al. Body dysmorphic disorder in different settings: A systematic review and estimated weighted prevalence. *Body Image* 2016;18:168-86.
13 Organization WH. The ICD-10 classification of mental and behavioural disorders: Clinical descriptions and diagnostic guidelines. 10 ed. Geneva: World Health Organization 1992.
14 Socialstyrelsen. Internationell statistisk klassifikation av sjukdomar och relaterade hälsoproblem – Systematisk förteckning, svensk version 2018 (ICD-10-SE). 10 ed 2020.
15 Phillips KA, Wilhelm S, Koran LM, et al. Body dysmorphic disorder: some key issues for DSM-V. *J Depress Anxiety* 2010;27(6):573-91.
16 World Health Organization. International classification of diseases for mortality and morbidity statistics 11 ed 2018.
17 Phillips KA, Stein DJ, Rauch SL, et al. Should an obsessive-compulsive spectrum grouping of disorders be included in DSM-V? *J Depress Anxiety* 2010;27(6):528-55.
18 Stein DJ, Kogan CS, Atmaca M, et al. The classification of Obsessive-Compulsive and Related Disorders in the ICD-11. *J Affect Disord* 2016;190:663-74.
19 DSM-5 Changes: Implications for Child Serious Emotional Disturbance. Table 23, DSM-IV to DSM-5 Body Dysmorphic Disorder Comparison. Substance Abuse and Mental Health Services Administration, Rockville, MD 2016. https://www.ncbi.nlm.nih.gov/books/NBK519712/table/ch3.t19/ (accessed 26 march 2021)
20 Impact of the DSM-IV to DSM-5 Changes on the National Survey on Drug Use and Health. Table 3.32, DSM-IV to DSM-5 Illness Anxiety Disorder Comparison. Substance Abuse and Mental Health Services Administration, Rockville, MD 2016. https://www.ncbi.nlm.nih.gov/books/NBK519704/table/ch3.t32/ (accessed 26 march 2021)
21 Busner J, Targum SD. The clinical global impressions scale: applying a research tool in clinical practice. *Psychiatry (Edgmont)* 2007;4(7):28-37.
22 Guy W. ECDEU assessment manual for psychopharmacology. Rockville, Md: US Dept. of Helath, Education and Welfare, Public Health Service, Alcohol, Drug Abuse and Mental Health Administration, National Institute of Mental Health, Pshychopharmacology, Research Branch, Division of Extramural Reserach Programs 1976.

For peer review only - http://bmjopen.bmj.com/site/about/guidelines.xhtml

23 Endicott J, Spitzer RL, Fleiss JL, et al. The global assessment scale. A procedure for measuring overall severity of psychiatric disturbance. *Arch Gen Psychiatry* 1976;33(6):766-71.
24 Leon AC, Shear MK, Klerman GL, et al. A comparison of symptom determinants of patient and clinician global ratings in patients with panic disorder and depression. *J Clin Psychopharmacol* 1993;13(5):327-31.
25 Jones SH, Thornicroft G, Coffey M, et al. A brief mental health outcome scale-reliability and validity of the Global Assessment of Functioning (GAF). *Br J Psychiatry* 1995;166(5):654-9.
26 Feinstein AR, Cicchetti DV. High agreement but low kappa: I. The problems of two paradoxes. *J Clin Epidemiol* 1990;43(6):543-9.
27 Shankar V, Bangdiwala SI. Observer agreement paradoxes in 2x2 tables: comparison of agreement measures. *BMC Med Res Methodol* 2014;14(1):100.
28 McHugh ML. Interrater reliability: the kappa statistic. *Biochem Med (Zagreb)* 2012;22(3):276-82.
29 Koo TK, Li MY. A Guideline of Selecting and Reporting Intraclass Correlation Coefficients for Reliability Research. *J Chiropr Med* 2016;15(2):155-63.
30 Landis JR, Koch GG. The measurement of observer agreement for categorical data. *Biometrics* 1977;33(1):159-74.
31 Barsky AJ, Ettner SL, Horsky J, et al. Resource utilization of patients with hypochondriacal health anxiety and somatization. *Med Care* 2001;39(7):705-15.
32 Bowyer L, Krebs G, Mataix-Cols D, et al. A critical review of cosmetic treatment outcomes in body dysmorphic disorder. *Body Image* 2016;19:1-8.
33 Phillips K, Didie ER, Menard W, et al. Clinical features of body dysmorphic disorder in adolescents and adults. *Psychiatry Res* 2006;141(3):305-14.
34 Fink P, Ørnbøl E, Christensen KS. The outcome of health anxiety in primary care. A two-year follow-up study on health care costs and self-rated health. *PLoS One* 2010;5(3):e9873.

For peer review only

For peer review only - http://bmjopen.bmj.com/site/about/guidelines.xhtml

TABLES AND FIGURES

Table 1. Alternative diagnoses for false positive cases of hypochondriasis (*n*=17) and dysmorphophobia (*n*=11).

**Hypochondriasis**	***n***	**Dysmorphophobia**	***n***
Dysmorphophobia	11	Excoriation (skin-picking) disorder	3
Psychotic disorder	4	Factitial dermatitis	3
Somatization disorder	2	Eating disorder, unspecified	3
Somatoform disorder, unspecified	2	Hypochondriasis	2
Obsessive-compulsive disorder	2	Psychotic disorder	2
Major depressive disorder	2	Pervasive developmental disorder	2
Borderline personality disorder	2	Delusional disorder	1
General anxiety disorder	1	Somatization disorder	1
Anxiety disorder, unspecified	1	Obsessive-compulsive disorder	1
Bipolar disorder	1	Trichotillomania	1
Pervasive developmental disorder	1	Social phobia	1
Substance dependence disorder	1	Generalized anxiety disorder	1
Acute stress reaction	1	Post-traumatic stress disorder	1
		Gender identity disorder	1
		Borderline personality disorder	1

Note: Numbers do not add up to the total of false positive cases (*n*=17 for hypochondriasis and *n*=11 for dysmorphophobia) since, for multiple cases, raters suggested more than one alternative diagnosis.

For peer review only

Figure 1. Flowchart of requested and received patient files containing a hypochondriasis (H) or a dysmorphophobia (D) diagnosis code.

Figure 2. Score distribution of the Clinical Global Impression – Severity (CGI-S) and Global Assessment of Functioning (GAF) by rater, for hypochondriasis (Panel A) and for dysmorphophobia (Panel B).

For peer review only

For peer review only - http://bmjopen.bmj.com/site/about/guidelines.xhtml

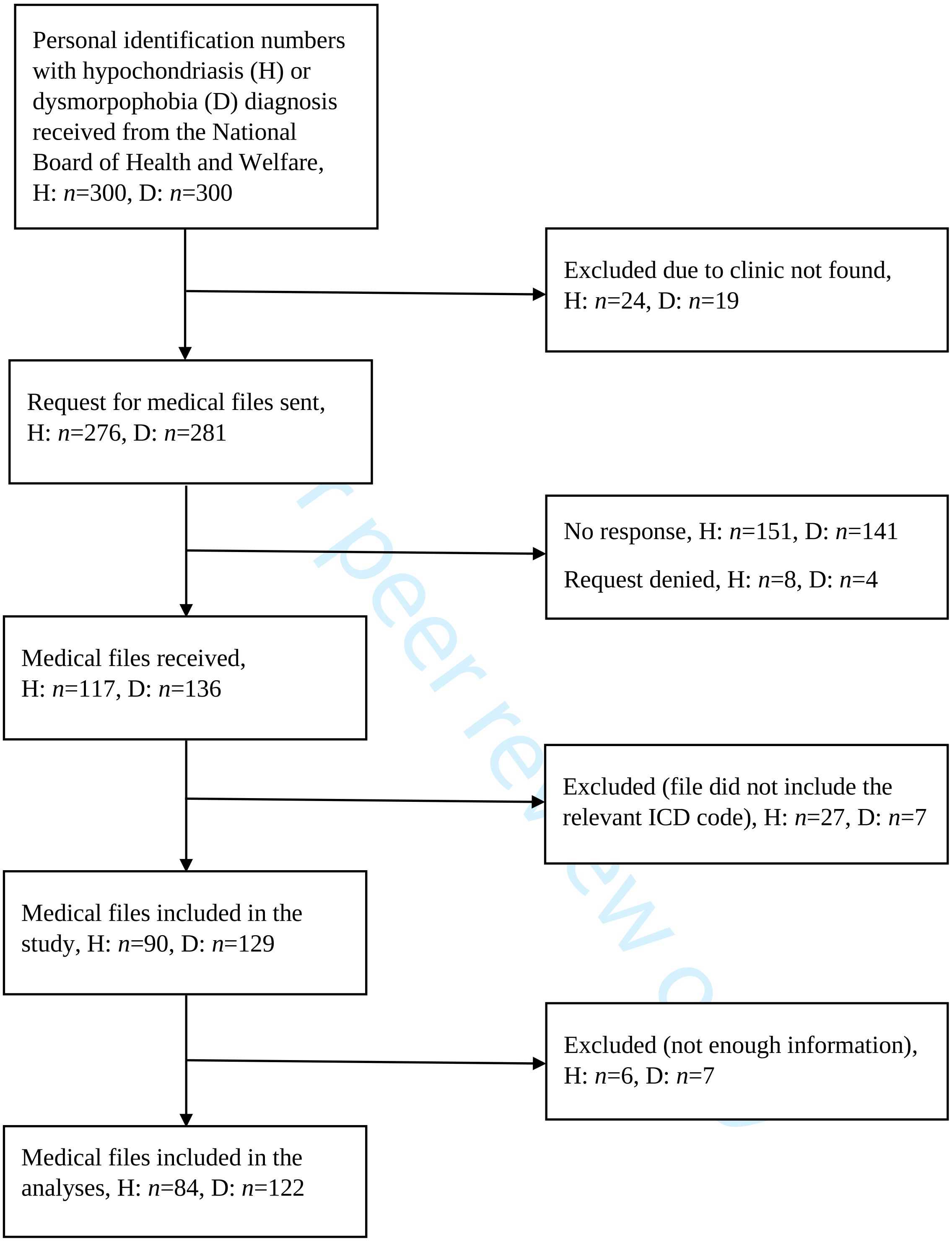
Personal identification numbers with hypochondriasis (H) or dysmorpophobia (D) diagnosis received from the National Board of Health and Welfare, H: n=300, D: n=300
Excluded due to clinic not found, H: n=24, D: n=19
Request for medical files sent, H: n=276, D: n=281
No response, H: n=151, D: n=141
Request denied, H: n=8, D: n=4
Medical files received, H: n=117, D: n=136
Excluded (file did not include the relevant ICD code), H: n=27, D: n=7
Medical files included in the study, H: n=90, D: n=129
Excluded (not enough information), H: n=6, D: n=7
Medical files included in the analyses, H: n=84, D: n=122

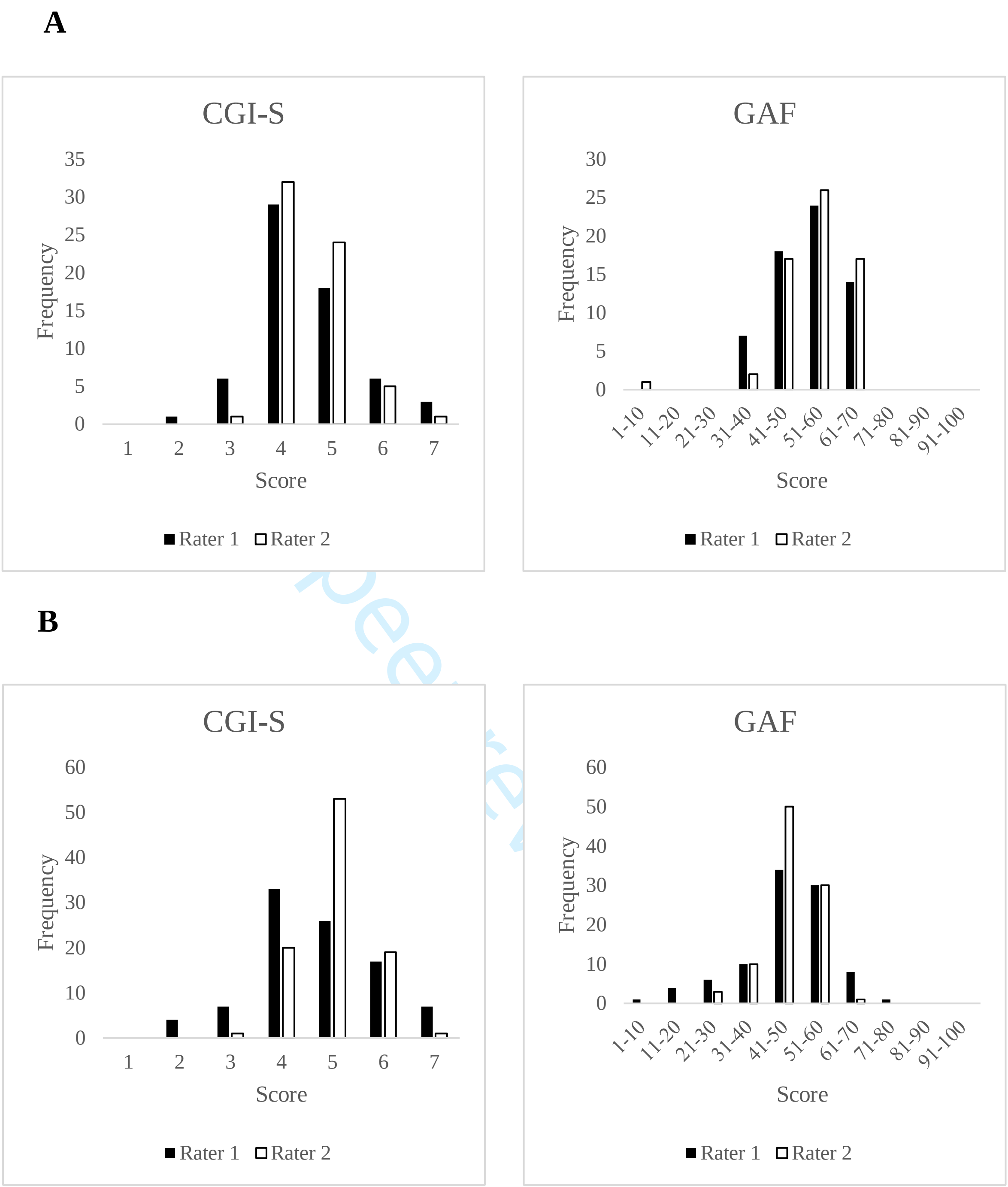
A
CGI-S
Frequency
35
30
25
20
15
10
5
0
1
2
3
4
5
6
7
Score
Rater 1
Rater 2
GAF
Frequency
30
25
20
15
10
5
0
1-10
11-20
21-30
31-40
41-50
51-60
61-70
71-80
81-90
91-100
Score
Rater 1
Rater 2
B
CGI-S
Frequency
60
50
40
30
20
10
0
1
2
3
4
5
6
7
Score
Rater 1
Rater 2
GAF
Frequency
60
50
40
30
20
10
0
1-10
11-20
21-30
31-40
41-50
51-60
61-70
71-80
81-90
91-100
Score
Rater 1
Rater 2

Supplementary material

Scoring sheet for the validation of Hypochondriasis and Dysmorphophobia codes

Rater: ____

Participant code: ______________________________ **Sex:** ◯ Man / ◯ Woman

Clinic: ◯ Psychiatry / ◯ Non-psychiatric; specialty: ______________________

ICD-10 definition of F45.2 hypochondrical disorder (which includes both hypochondriasis and dysmorphophobia)

Hypochondriacal disorder – The essential feature is a persistent preoccupation with the possibility of having one or more serious and progressive physical disorders. Patients manifest persistent somatic complaints **or** a persistent preoccupation with their physical appearance. Normal or commonplace sensations and appearances are often interpreted by patients as abnormal and distressing, and attention is usually focused upon only one or two organs or systems of the body. Marked depression and anxiety are often present, and may justify additional diagnoses.

Please tick in the right column the code that better describes the clinical presentation:

**Hypochondria**, if persistent somatic complaints	
**Dysmorphophobia**, if persistent preoccupation with their physical appearance	

DSM-IV-TR criteria for Hypochondriasis (tick those that apply on the right column)

**A.** Preoccupation with fears of having, or the idea that one has, a serious disease based on the person's misinterpretation of bodily symptoms.	
**B.** The preoccupation persists despite appropriate medical evaluation and reassurance.	
**C.** The belief in Criterion A is not of delusional intensity (as in Delusional Disorder, Somatic Type) and is not restricted to a circumscribed concern about appearance (as in Body Dysmorphic Disorder).	
**D.** The preoccupation causes clinically significant distress or impairment in social, occupational, or other important areas of functioning.	
**E.** The duration of the disturbance is at least 6 months.	
**F.** The preoccupation is not better accounted for by Generalized Anxiety Disorder, Obsessive-Compulsive Disorder, Panic Disorder, a Major Depressive Episode, Separation Anxiety, or another Somatoform Disorder.	

DSM-5 criteria for Illness Anxiety Disorder (tick those that apply on the right column)

**A.** Preoccupation with having or acquiring a serious illness.	
**B.** Somatic symptoms are not present or, if present, are only mild in intensity. If another medical condition is present or there is a high risk for developing a medical condition (e.g., strong family history is present), the preoccupation is clearly excessive or disproportionate.	

For peer review only - http://bmjopen.bmj.com/site/about/guidelines.xhtml

**C.** There is a high level of anxiety about health, and the individual is easily alarmed about personal health status.	
**D.** The individual performs excessive health-related behaviors (e.g., repeatedly checks his or her body for signs of illness) or exhibits maladaptive avoidance (e.g., avoids doctor appointments and hospitals).	
**E.** Illness preoccupation has been present for at least 6 months, but the specific illness that is feared may change over that period of time.	
**F.** The illness-related preoccupation is not better explained by another mental disorder, such as somatic symptom disorder, panic disorder, generalized anxiety disorder, body dysmorphic disorder, obsessive-compulsive disorder, or delusional disorder, somatic type.	

DSM-IV-TR criteria for Body Dysmorphic Disorder (tick those that apply on the right column)

**A.** Preoccupation with an imagined defect in appearance. If a slight physical anomaly is present, the person's concern is markedly excessive.	
**B.** The preoccupation causes clinically significant distress or impairment in social, occupational, or other important areas of functioning.	
**C.** The preoccupation is not better accounted for by another mental disorder (e.g., dissatisfaction with body shape and size in anorexia nervosa).	

DSM-5 criteria for Body Dysmorphic Disorder (tick those that apply on the right column)

**A.** Preoccupation with one or more perceived defects or flaws in physical appearance that are not observable or appear slight to others.	
**B.** At some point during the course of the disorder, the individual has performed repetitive behaviors (e.g., mirror checking, excessive grooming, skin picking, reassurance seeking) or mental acts (e.g., comparing his or her appearance with that of others) in response to the appearance concerns.	
**C.** The preoccupation causes clinically significant distress or impairment in social, occupational, or other important areas of functioning.	
**D.** The appearance preoccupation is not better explained by concerns with body fat or weight in an individual whose symptoms meet diagnostic criteria for an eating disorder.	

Assessment

Diagnosis. Please mark all the answers that apply.

Hypochondria	Body Dysmorphic Disorder
◯ Diagnostic criteria are clearly met, according to the ICD-10 definition	◯ Diagnostic criteria are clearly met, according to the ICD-10 definition
◯ Diagnostic criteria are clearly met, according to DSM-IV-TR criteria	◯ Diagnostic criteria are clearly met, according to DSM-IV-TR criteria
◯ Diagnostic criteria are clearly met, according to DSM-5 criteria	◯ Diagnostic criteria are clearly met, according to DSM-5 criteria
◯ Probable, according to the ICD-10 definition	◯ Probable, according to the ICD-10 definition
◯ Probable, according to DSM-IV-TR criteria	◯ Probable, according to DSM-IV-TR criteria
◯ Probable, according to DSM-5 criteria	◯ Probable, according to DSM-5 criteria

◯ Neither ICD-10 nor DSM-IV criteria for **Hypochondria** nor **Body Dysmorphic Disorder** are met

◯ Insufficient information to make a decision about either **Hypochondria** or **Body Dysmorphic Disorder** diagnosis because there is no or very minimal description of psychiatric symptoms in the file (e.g., the file comes from a non-psychiatric clinic with no description of psychiatric symptoms, only blood tests available, etc.)

If neither Hypochondria nor Body Dysmorphic Disorder are present, please state the most likely alternative:

____

____

Clinical Global Impression – Severity (CGI-S). Considering your total clinical experience with this particular population, how mentally ill is the patient? (refer to the hypochondria or body dysmorphic symptoms only; if they are not present, leave this question blank)

0. ◯ Cannot be assessed (insufficient information)
1. ◯ Normal, not at all ill
2. ◯ Borderline mentally ill
3. ◯ Mildly ill
4. ◯ Moderately ill
5. ◯ Markedly ill
6. ◯ Severely ill
7. ◯ Among the most extremely ill patients

Global Assessment of Functioning (GAF) Scale. Consider psychological, social, and occupational functioning on a hypothetical continuum of mental health-illness. Do not include impairment in functioning due to physical (or environmental) limitations.

Please write your score from 0 to 100 *(consult the attached GAF scale for reference)* ☐

For peer review only

STROBE Statement—checklist of items that should be included in reports of observational studies

	Item No.	Recommendation	Reported on Page Number/Line Number	Reported on Section/Paragraph
**Title and abstract**	1	(*a*) Indicate the study's design with a commonly used term in the title or the abstract	Page 1, line 19 / Page 2, line 21	Title page / Abstract
		(*b*) Provide in the abstract an informative and balanced summary of what was done and what was found	Page 2, line 23-58 + Page 3, line 3-6	Abstract
**Introduction**				
Background/rationale	2	Explain the scientific background and rationale for the investigation being reported	Page 4, line 29-60 + Page 5, line 3-13	Introduction, Paragraph 2
Objectives	3	State specific objectives, including any prespecified hypotheses	Page 5, line 15-20	Introduction, Paragraph 3
**Methods**				
Study design	4	Present key elements of study design early in the paper	Page 6, line 44-49	Methods, Paragraph 4
Setting	5	Describe the setting, locations, and relevant dates, including periods of recruitment, exposure, follow-up, and data collection	Page 5, line 31-60 + Page 6, line 3-6	Methods, Paragraph 1-2
Participants	6	(*a*) *Cohort study*—Give the eligibility criteria, and the sources and methods of selection of participants. Describe methods of follow-up<br>*Case-control study*—Give the eligibility criteria, and the sources and methods of case ascertainment and control selection. Give the rationale for the choice of cases and controls<br>*Cross-sectional study*—Give the eligibility criteria, and the sources and methods of selection of participants	Page 5, line 31-60 + Page 6, line 3-25	Methods, Paragraph 1-3
		(*b*) *Cohort study*—For matched studies, give matching criteria and number of exposed and unexposed	Not applicable to our paper	Not applicable to our paper

For peer review only - http://bmjopen.bmj.com/site/about/guidelines.xhtml

		Case-control study—For matched studies, give matching criteria and the number of controls per case		
Variables	7	Clearly define all outcomes, exposures, predictors, potential confounders, and effect modifiers. Give diagnostic criteria, if applicable	Page 5, line 47-60 + Page 6, line 3-6 + Page 7, line 33-59 + Page 8, line 3-11	Methods, Paragraph 2, 6
Data sources/ measurement	8*	For each variable of interest, give sources of data and details of methods of assessment (measurement). Describe comparability of assessment methods if there is more than one group	Page 6, line 44-59 + Page 7, line 3-59 + Page 8, line 3-11	Methods, Paragraph 4-6
Bias	9	Describe any efforts to address potential sources of bias	Page 5, line 38-45	Methods, Paragraph 1
Study size	10	Explain how the study size was arrived at	Page 6, line 8-11	Methods, Paragraph 3

Continued on next page

For peer review only - http://bmjopen.bmj.com/site/about/guidelines.xhtml

Quantitative variables	11	Explain how quantitative variables were handled in the analyses. If applicable, describe which groupings were chosen and why	Page 8, line 18-57	Methods, Paragraph 7-9
Statistical methods	12	(*a*) Describe all statistical methods, including those used to control for confounding	Page 8, line 18-57	Methods, Paragraph 7-9
		(*b*) Describe any methods used to examine subgroups and interactions	Not applicable	Not applicable
		(*c*) Explain how missing data were addressed		
		(*d*) *Cohort study*—If applicable, explain how loss to follow-up was addressed<br>*Case-control study*—If applicable, explain how matching of cases and controls was addressed<br>*Cross-sectional study*—If applicable, describe analytical methods taking account of sampling strategy	Not applicable as this was a retrospective chart review	Not applicable as this was a retrospective chart review
		(*e*) Describe any sensitivity analyses	-	-
**Results**				
Participants	13*	(a) Report numbers of individuals at each stage of study—eg numbers potentially eligible, examined for eligibility, confirmed eligible, included in the study, completing follow-up, and analysed	Page 20, line 3-49	Figure 1
		(b) Give reasons for non-participation at each stage	Page 20, line 3-49	Figure 1
		(c) Consider use of a flow diagram	Page 20, line 3-49	Figure 1
Descriptive data	14*	(a) Give characteristics of study participants (eg demographic, clinical, social) and information on exposures and potential confounders	Page 9, line 26-34 + Page 10, line 21-29	Results, Paragraph 1, 5
		(b) Indicate number of participants with missing data for each variable of interest	Page 11, line 16-59	Results, Paragraph 9-10
		(c) *Cohort study*—Summarise follow-up time (eg, average and total amount)	Not applicable	Not applicable
Outcome data	15*	*Cohort study*—Report numbers of outcome events or summary measures over time	Not applicable	Not applicable

For peer review only - http://bmjopen.bmj.com/site/about/guidelines.xhtml

		Case-control study—Report numbers in each exposure category, or summary measures of exposure	Not applicable	Not applicable
		Cross-sectional study—Report numbers of outcome events or summary measures	Not applicable	Not applicable
Main results	16	(*a*) Give unadjusted estimates and, if applicable, confounder-adjusted estimates and their precision (eg, 95% confidence interval). Make clear which confounders were adjusted for and why they were included	Page 9, line 36-60 + Page 10, line 3-13, 30-60 + Page 11, line 3-59	Results, Paragraph 2-4, 6-10
		(*b*) Report category boundaries when continuous variables were categorized	-	-
		(*c*) If relevant, consider translating estimates of relative risk into absolute risk for a meaningful time period	-	-

Continued on next page

For peer review only - http://bmjopen.bmj.com/site/about/guidelines.xhtml

Other analyses	17	Report other analyses done—eg analyses of subgroups and interactions, and sensitivity analyses	-	-
**Discussion**				
Key results	18	Summarise key results with reference to study objectives	Page 13, line 9-30	Discussion, Paragraph 1
Limitations	19	Discuss limitations of the study, taking into account sources of potential bias or imprecision. Discuss both direction and magnitude of any potential bias	Page 14, line 3-29	Discussion, Paragraph 5
Interpretation	20	Give a cautious overall interpretation of results considering objectives, limitations, multiplicity of analyses, results from similar studies, and other relevant evidence	Page 12, line 13-60 + Page 13, line 3-8	Discussion, Paragraph 1-2
Generalisability	21	Discuss the generalisability (external validity) of the study results	Page 13, line 10-30	Discussion, Paragraph 3
**Other information**				
Funding	22	Give the source of funding and the role of the funders for the present study and, if applicable, for the original study on which the present article is based	Page 14, line 52-57	Below Conclusions

*Give information separately for cases and controls in case-control studies and, if applicable, for exposed and unexposed groups in cohort and cross-sectional studies.

Note: An Explanation and Elaboration article discusses each checklist item and gives methodological background and published examples of transparent reporting. The STROBE checklist is best used in conjunction with this article (freely available on the Web sites of PLoS Medicine at http://www.plosmedicine.org/, Annals of Internal Medicine at http://www.annals.org/, and Epidemiology at http://www.epidem.com/). Information on the STROBE Initiative is available at www.strobe-statement.org.

For peer review only
